# Microstates and power envelope hidden Markov modeling probe bursting brain activity at different timescales

**DOI:** 10.1016/j.neuroimage.2021.118850

**Published:** 2022-02-15

**Authors:** N. Coquelet, X. De Tiège, L. Roshchupkina, P. Peigneux, S. Goldman, M. Woolrich, V. Wens

**Affiliations:** a Laboratoire de Cartographie fonctionnelle du Cerveau (LCFC), UNI – ULB Neuroscience Institute, Université libre de Bruxelles, Brussels 1070, Belgium; b Magnetoencephalography Unit, Service of Translational Neuroimaging, CUB – Hôpital Erasme, Brussels, Belgium; c Neuropsychology and Functional Neuroimaging Research Unit (UR2NF), Centre for Research in Cognition and Neurosciences (CRCN), UNI – ULB Neuroscience Institute, Université libre de Bruxelles, Brussels, Belgium; d Oxford Centre for Human Brain Activity, Wellcome Centre for Integrative Neuroimaging, Department of Psychiatry, University of Oxford, Oxford, United Kingdom

**Keywords:** Electroencephalography, Magnetoencephalography, Power bursts, Resting state, State classification

## Abstract

State modeling of whole-brain electroencephalography (EEG) or magnetoencephalography (MEG) allows to investigate transient, recurring neurodynamical events. Two widely-used techniques are the microstate analysis of EEG signals and hidden Markov modeling (HMM) of MEG power envelopes. Both reportedly lead to similar state lifetimes on the 100 ms timescale, suggesting a common neural basis. To investigate whether microstates and power envelope HMM states describe the same neural dynamics, we used simultaneous MEG/EEG recordings at rest and compared the spatial signature and temporal activation dynamics of microstates and power envelope HMM states obtained separately from EEG and MEG. Results showed that microstates and power envelope HMM states differ both spatially and temporally. Microstates reflect sharp events of neural synchronization, whereas power envelope HMM states disclose network-level activity with 100–200 ms lifetimes. Further, MEG microstates do not correspond to the canonical EEG microstates but are better interpreted as split HMM states. On the other hand, both MEG and EEG HMM states involve the (de)activation of similar functional networks. Microstate analysis and power envelope HMM thus appear sensitive to neural events occurring over different spatial and temporal scales. As such, they represent complementary approaches to explore the fast, sub-second scale bursting electrophysiological dynamics in spontaneous human brain activity.

## 1 Introduction

A fundamental part of human neural dynamics is the spontaneous emergence of brain rhythms, i.e., large-scale oscillations of neuroelectric activity (for a review, see, e.g., (Hari and Salmelin, 1997)). These rhythms play a critical role for human brain functions such as sensory, motor and cognitive processes ((Klimesch, 2012), for reviews, see (Klimesch et al., 2010; Pfurtscheller and Lopes da Silva, 1999)). They also wax and wane spontaneously at rest (i.e., in the absence of any explicit task performance). The resulting fluctuations in their amplitude are key to intrinsic functional brain connectivity (Siegel et al., 2012). When measured with electroencephalography (EEG) or magnetoencephalography (MEG), this oscillatory dynamics leads to signal power time courses whose correlation structure identifies functional brain networks (Brookes et al., 2011; Coquelet et al., 2020a; Hipp et al., 2012; Liu et al., 2017; Siems et al., 2016; Wens et al., 2014). Further, spontaneous MEG/EEG power fluctuations occur in transient, sub-second long bursts of oscillatory activity (van Ede et al., 2018). Short-lived power bursts may actually correspond to the fast activation/deactivation of functional networks (Baker et al., 2014; Britz et al., 2010; Vidaurre et al., 2018) and their co-occurrence, to the intrinsic functional connectivity of these networks (Seedat et al., 2020). They might ultimately relate to the metastable cross-network interactions characteristic of functional integration at the supra-second timescale (de Pasquale et al., 2016, 2012; Della Penna et al., 2019; Wens et al., 2019). Power bursts also presumably hold specific functions, such as the encoding of recently acquired information by coactivation with spontaneous replays (Higgins et al., 2020). Exploring the spontaneous dynamics of MEG/EEG power bursts thus represents a fundamental step towards a better understanding of the functional architecture of the human brain at rest.

With their millisecond-scale temporal resolution, EEG and MEG (Hari and Puce, 2017) are natural techniques to investigate power bursts, although the role of short-time events has also been emphasized with functional magnetic resonance imaging (fMRI) (Tagliazucchi et al., 2012). Accordingly, the two main data-driven methods used to detect recurring events of high electrophysiological power are EEG microstate analysis ((Lehmann et al., 1987); for a review, see (Michel and Koenig, 2018)) and hidden Markov modeling (HMM) of MEG power envelopes (Baker et al., 2014; Quinn et al., 2018). Both allow to partition EEG/MEG data into discrete brain states that recurrently activate and deactivate one after the other, yet the underlying clustering algorithms strongly differ in their assumptions and methods. Microstates are determined as time periods of quasi-stable scalp EEG topography that repeatedly occur, up to amplitude rescalings and polarity flips. Four canonical microstates have been identified with reported mean lifetimes ranging from 60 to 120 ms (for a review, see, e.g., (Michel and Koenig, 2018)). These microstates were associated with different classes of mentation (Lehmann et al., 1998) and partially correlated with the spontaneous haemodynamics of some fMRI networks (Britz et al., 2010; Musso et al., 2010; Yuan et al., 2012). Their temporal properties are also affected by brain disorders such as schizophrenia (Koenig et al., 1999; Lehmann et al., 2005) or multiple sclerosis (Gschwind et al., 2016). By contrast, the HMM relies on the more abstract concept of Markov chains to describe brain power dynamics in terms of causal transitions among “hidden” states (Rabiner, 1989). These states are hidden in the sense that they are not explicitly expressed in the data and must be inferred through implicit statistical features such as, e.g., the covariance matrix of a state observation model (Rezek and Roberts, 2005). Here, the HMM states are determined by transient patterns of MEG power envelope covariance repeating over time (Baker et al., 2014), but occurring on too short time periods to be measurable directly from the data, e.g., with sliding windows (for a review, see (O'Neill et al., 2018)). The HMM inference applied to MEG power envelope signals has typically been used to identify 6 or 8 states disclosing a spatial distribution reminiscent of brain functional networks as well as mean lifetimes ranging from 50 to 200 ms (Baker et al., 2014; Quinn et al., 2018). Temporal properties of HMM states are also altered by physiological processes such as aging (Brookes et al., 2018; Coquelet et al., 2020b) as well as brain disorders such as Alzheimer's disease (Puttaert et al., 2020; Sitnikova et al., 2018) or multiple sclerosis (Van Schependom et al., 2019).

Interestingly, despite fundamental methodological differences, EEG microstates and MEG power envelope HMM states appear to remain stable over similar timescales. This raises the question of whether they describe similar neural dynamics ((Baker et al., 2014); for a review, see (Khanna et al., 2015)). Here, we investigate this key question using simultaneous MEG/EEG recordings of resting-state activity. We started by directly comparing the spatial topography and temporal dynamics of EEG microstates as classically classified from EEG electrode signals (Michel and Koenig, 2018), and of MEG power envelope HMM states as classically inferred from brain source activity reconstructed from MEG sensor signals (Baker et al., 2014). Still, such direct comparison entangles several effects and might leave the origin of potential differences undetermined, since it mixes the impact of the state clustering model itself (microstates vs. HMM), the recording modality (EEG vs. MEG), the type of signal inputted to the clustering model (scalp recordings vs. source-reconstructed brain activity), as well as several other parameters (e.g., signal filters and the number of states to be classified). Of particular interest is to assess the effect of the state clustering model itself, all other parameters being fixed. Our strategy to do so was to feed the exact same signals to both microstate and HMM state classification algorithms, so confounds related to recording modality or processing parameters are avoided. This approach required adapting the classical notion of microstate to MEG, and that of HMM state to EEG. To the best of our knowledge, a microstate analysis of MEG data has not yet been developed. The HMM approach has been applied to EEG power envelopes (Hunyadi et al., 2019; Sitnikova et al., 2020), but the focus was on the relationship with fMRI networks rather than microstates. We also considered here an application of power envelope HMM to MEG/EEG sensor signals (rather than MEG source signals as originally done in (Baker et al., 2014) and in our first, direct comparison) to further disentangle the impact of source reconstruction on state classification. In addition, this setup provides an opportunity to establish how recording modality affects microstates and HMM states, which allows us to extend a previous comparative study of MEG and EEG resting-state signals (Coquelet et al., 2020a). Finally, several other parameters were varied in order to assess their importance in microstate and HMM state classification.

Our main objective was therefore to compare microstates and HMM states, and to identify the specific impact of both the state clustering model and the recording modality on temporal and spatial signatures of transient brain states. To do so, we estimated to what extent two types of states tend to co-activate by temporal correlation analysis of their activation dynamics, and to what extent they involve similar brain regions or networks by spatial correlation analysis of the associated power distributions. Based on the idea that microstates and HMM states are both designed to identify discrete recurrent brain states and given the reported similarity of their typical lifetimes (Baker et al., 2014), we expected to identify similar features. In particular, we hypothesized that the two state clustering models would reveal a close spatio-temporal relationship within each recording modality. This would suggest that microstates and power envelope HMM states disclose similar neural events. On the other hand, based on a previous comparison of MEG and EEG power envelope signals at rest (Coquelet et al., 2020a), we expected similar spatial signatures but substantially different temporal state dynamics across the two recording modalities.

## 2 Methods

### 2.1 Participants

Forty-two young adults (14 females, mean age ± standard deviation (SD): 24.4 ± 3.9 years, range: 18–35 years) were included in this study, 19 of which were already used in a previous study of our group (Coquelet et al., 2020a). All participants were right-handed according to the Edinburgh handedness inventory (Oldfield, 1971), did not take any psychotropic drug, and had no prior history of neurological or psychiatric disorder. Each of them signed a written informed consent before scanning. The CUB – Hôpital Erasme Ethics Committee approved this study prior to their inclusion.

### 2.2 Data acquisition

Participants underwent a resting-state recording session (eyes open, fixation cross, 5 min) with simultaneous MEG and high-density EEG. Neuromagnetic activity was recorded with a 306-channel whole-scalp MEG system (band-pass: 0.1–330 Hz, sampling frequency: 1 kHz) installed in a light-weight magnetically shielded room (Maxshield™, MEGIN, Helsinki, Finland; see (De Tiège et al., 2008) for detailed characteristics). Four coils continuously tracked subjects’ head position inside the MEG helmet. The first 15 participants were scanned with a Neuromag Vectorview™ MEG (Elekta Oy, Helsinki, Finland) and the other 27 with a Neuromag Triux™ MEG (MEGIN, Helsinki, Finland) due to a system upgrade. These neuromagnetometers have identical sensor layout (i.e., 102 magnetometers and 102 pairs of orthogonal planar gradiometers) and only differ in sensor dynamic range and background magnetic environment, neither of which substantially affect data quality after preprocessing. In particular, previous research mixing resting-state recordings from these two systems did not disclose any significant difference (Coquelet et al., 2020b, 2020a; Naeije et al., 2020; Sjøgård et al., 2020).

Neuroelectric activity was measured with a MEG-compatible, 256-channel scalp EEG system (low-pass: 450 Hz; sampling frequency: 1 kHz) based on low profile, silver chloride-plated carbon-fiber electrode pellets (MicroCel Geodesic Sensor Net, Electrical Geodesics Inc., Magstim EGI, Eugene, Oregon, USA). The reference electrode was placed at Cz and all impedances were kept below 50 kΩ by application of a conductive gel between each electrode and the skin. Of note, good EEG signal quality is maintained despite allowing for such high impedances (compared to other EEG systems, see (Kappenman and Luck, 2010)) thanks to the usage of a high input-impedance amplifier (Net Amp GES 400, Electrical Geodesics Inc., Magstim EGI, Eugene, Oregon, USA), which eases subject preparation and avoids the need for skin abrasion (Ferree et al., 2001). A 100-ms long square-pulse trigger signal was generated by the MEG system electronics every second and fed to the EEG amplifier in order to enable clock synchronization of both systems. The location of the head position indicator coils, scalp EEG electrodes, and approximately 200 scalp points were determined with respect to anatomical fiducials using an electromagnetic tracker (Fastrack, Polhemus, Colchester, Vermont, USA).

Participant's high-resolution 3D T1-weighted cerebral magnetic resonance images (MRIs) were acquired on a 1.5 T MRI scanner (Intera, Philips, The Netherlands) after the MEG/EEG recordings.

### 2.3 Data preprocessing and source projection

The MEG data were preprocessed using signal space separation (Taulu et al., 2005) to subtract environmental magnetic noise and correct for head movements (Maxfilter v2.1, Elekta Oy, Helsinki, Finland). No bad channels were detected in the process. For EEG data, we started by eliminating 84 electrodes placed on cheeks and neck as they often suffered from excessive muscle artefacts or poor skin contact, leaving 172 scalp-matched electrodes. Remnant bad channels were then automatically detected and removed using artifact subspace reconstruction (Kothe and Makeig, 2013) as implemented in EEGLAB ((Delorme and Makeig, 2004); EEGLAB v2019.0, https://sccn.ucsd.edu/eeglab/index.php) (number of bad channels: 11 ± 4 out of 172, range: 4–21). Cardiac, ocular and remaining system artifacts were further eliminated from MEG and EEG data separately, using an independent component analysis of band-passed (1–40 Hz) signals ((Vigário et al., 2000); FastICA v2.5, http://www.cis.hut.fi/projects/ica/fastica, with dimension reduction to 30 components, symmetric approach, and cubic nonlinearity contrast). Artefactual components were identified by visual inspection and regressed out of the full-rank data (number of components removed for MEG: 4 ± 1, range: 2–7; for EEG: 14 ± 3, range: 9–21). Bad EEG electrodes were subsequently reconstructed using spherical spline interpolation (Perrin et al., 1989) and EEG scalp topographies were spatially filtered (Michel and Brunet, 2019) to remove any last local outlier. The resulting EEG data were then re-referenced to the average across the 172 scalp electrodes. The rank of the fully preprocessed data was 53 ± 3 (mean ± SD, range: 47–59) for MEG and 18 ± 5 (range: 10–35) for EEG. Finally, the synchronization of MEG and EEG signals was ensured by temporal realignment based on the trigger signal.

Separate forward models for MEG and EEG were computed based on the participants’ MRI, segmented beforehand using the FreeSurfer software ((Fischl, 2012); FreeSurfer v6.0; Martinos Center for Biomedical Imaging, Massachusetts, USA; https://surfer.nmr.mgh.harvard.edu, freesurfer-x86_64-linux-gnu-stable6–20,170,118). The coordinate systems of MEG and EEG were co-registered to the MRI coordinate system using the three anatomical fiducials for initial estimation and the head-surface points to manually refine the surface co-registration (MRIlab, MEGIN Data Analysis Package 3.4.4, MEGIN, Helsinki, Finland). The source space was built by placing three orthogonal current dipoles at each point of a grid derived from a regular 5-mm grid cropped within the Montreal Neurological Institute (MNI) template MRI volume and non-linearly deformed onto each participant's MRI with the Statistical Parametric Mapping software ((Friston et al., 2007); SPM12, Wellcome centre for Neuroimaging, London, UK; https://www.fil.ion.ucl.ac.uk/spm). Forward models were then computed on this source space using the one-layer boundary element method (BEM) for MEG and the three-layer BEM with default conductivity values for EEG (as used and discussed in (Coquelet et al., 2020a)) implemented in the MNE-C suite ((Gramfort et al., 2014); MNE-C v2.7.3, Martinos Center for Biomedical Imaging, Massachusetts, USA; https://mne.tools/stable/index.html). The EEG forward models were also re-referenced to their average across the 172 scalp electrodes.

Finally, neural source activity of MEG or EEG signals were reconstructed using minimum norm estimation (MNE, Dale and Sereno, 1993) as regularized inverse that allows to project the MEG (gradiometers only, see (Garcés et al., 2017)) or the EEG signals onto the 5 mm, dipolar source grid associated to the corresponding forward model. The noise covariance matrix was estimated individually on the basis of 5 min of empty-room data for MEG (with signal space separation), and as the identity projected in the sensor subspace corresponding to the average reference for EEG. The regularization parameter was estimated from the consistency condition derived in (Wens et al., 2015). Each three-dimensional dipole time series was projected onto the direction of maximum variance.

### 2.4 Microstate clustering

Microstate inference from EEG data followed standard steps (for reviews, see, e.g., (Khanna et al., 2015; Michel et al., 2009; Michel and Koenig, 2018)) and was performed using the EEGLAB plugin for microstate analysis (v1.1, http://www.thomaskoenig.ch/index.php/software/microstates-in-eeglab). Microstates were built from wideband filtered (4–30 Hz) EEG signals, but we also report on the effect of widening this band to 1–40 Hz (supplementary material S1) that is often used in the microstate literature (e.g., (Britz et al., 2010; Gschwind et al., 2016; Tomescu et al., 2015)). Sensor signals were then downsampled at 200 Hz with low-pass filtering at 100 Hz (Khanna et al., 2015; Michel et al., 2009; Michel and Koenig, 2018) using moving-window averaging. In specific comparisons of state clustering models and recording modalities, sensor signals were also downsampled at 40 Hz with low-pass at 10 Hz using moving-window averaging, similarly to the power envelope signals inputted to the HMM (see below).

We also adapted microstate classification to MEG. The main difference is that we focused on planar gradiometers as they disclose the highest signal-to-noise ratio (Hari and Puce, 2017) and combined each pair of orthogonal sensors using their Euclidean norm.

The first step of the microstate analysis consists in a two-level clustering of time-varying sensor topographies in order to define the spatial signature of each microstate. Atomize-agglomerate hierarchical clustering (AAHC; (Tibshirani and Walther, 2005)) was used to partition each individual dataset into a number *K* of prototypal topographical maps determined so as to maximize spatial variance, a.k.a. global field power (GFP). Briefly, AAHC starts from instantaneous sensor maps and iteratively builds clusters by breaking one cluster into its constituent maps (atomization) and reassigning each of them to the cluster whose topography best fits theirs in terms of absolute spatial correlation (agglomeration). In this algorithm, the topography associated to a cluster is defined as the principal component of its constituent maps, and the cluster to atomize at each iteration is chosen deterministically as the one with least GFP. This procedure ensures that microstates are explicitly geared towards the detection of recurring patterns of highest GFP. The number *K* of clusters was merely fixed to *K* = 4 as this value is largely representative of the literature ((Koenig et al., 1999); for a review, see (Michel and Koenig, 2018)). However, such prior may represent a limitation (which is why data-driven selection techniques are increasingly recommended; for a review, see (Murray et al., 2008)), so we further checked the robustness of our results by considering the case *K* = 6 as well (see below). The resulting set of individual-level topographies were then subjected to a full permutation procedure (Koenig et al., 1999) in order to obtain the final group-level microstate topographies.

It is noteworthy that this two-level clustering approach is common in the microstate literature but differs from the group HMM approach (see below), so for better comparability we also considered a “group AAHC” applied to instantaneous spatial maps across all subjects at once (supplementary material S2). Also noteworthy is the fact that AAHC is restricted to time points corresponding to local maxima of the GFP time series in order to reduce computational complexity (Khanna et al., 2015). Since by design the HMM does not involve such subselection of time points, we applied microstate clustering to the unrestricted, continuous signals as well (supplementary material S3).

The second step consists in obtaining a binary time series of microstate activation/inactivation. We defined here these time series using the criterion that the microstate active at any given time point be the one whose topography best fits (again in terms of absolute spatial correlation) the instantaneous sensor topography at this time point (Brunet et al., 2011). Microstate activation is thus *exclusive*, i.e., two microstates cannot be simultaneously active, and *complete*, i.e., a microstate is active at any time. Importantly, this basic criterion is fairly close in spirit to the Viterbi algorithm used in the HMM (as explained below), but it is frequently altered in the microstate literature by using a temporally smoothed version of these binary time series (da Cruz et al., 2020; D'Croz-Baron et al., 2019; Krylova et al., 2020; Pascual-Marqui et al., 1995; Sikka et al., 2020). In our direct comparison, we implemented the popular approach whereby microstate activation is determined as described above but at GFP peaks only and is then extended between these peaks by nearest-neighbor interpolation (Krylova et al., 2020; Sikka et al., 2020). The Viterbi algorithm does not involve such *ad-hoc* temporal smoothing, but at the same time the HMM itself intrinsically enforces a degree of causality, and thus temporal smoothness, in state time courses. For this reason, we also considered the raw (non-interpolated) microstate time series and examined the effect of temporal smoothing on microstate activation dynamics (supplementary material S4).

### 2.5 Hidden Markov modeling of power envelopes

The HMM was inferred from power envelope time courses estimated by Hilbert transformation of wideband filtered (4–30 Hz) signals, using the GLEAN toolbox (GLEAN0.3, https://github.com/OHBA-analysis/GLEAN) originally developed for and applied to MEG source power (Baker et al., 2014). The focus on continuous power envelopes makes the HMM analysis geared towards the detection of power bursts (van Ede et al., 2018). In our direct comparison, we considered HMM states inferred from power envelope MEG source signals reconstructed with MNE (Coquelet et al., 2020b). In our specific comparisons assessing the impact of state clustering or recording modality, the HMM inference was also done directly at the sensor level by adapting the aforementioned pipeline to both MEG gradiometer and EEG power envelopes.

Individual datasets of power envelope signals were downsampled at 40 Hz with low-pass filtering at 10 Hz via moving-window averaging, demeaned and normalized by the global variance across sensors, concatenated temporally across subjects to design a group-level analysis, and finally projected onto their *N* first principal components for dimensionality reduction prior to HMM inference (Baker et al., 2014). For MEG source power envelopes, we used *N* = 53, which retains about 55% of variance. For sensor-level MEG and EEG power envelope signals, the dimension reduction was chosen so as to explain a comparable fraction of variance across MEG and EEG sensor data. Specifically, *N* = 10 components were retained for EEG and *N* = 41 for MEG, which corresponded to 81% of explained variance in both cases. Such low dimensionality for EEG presumably relies on the high spatial smoothness of EEG (see, e.g., (Coquelet et al., 2020a)). So this approach takes into account the intrinsic difference in spatial smoothness of MEG and EEG. (See however supplementary material S5 for a version of the EEG power envelope HMM with *N* = 41 instead, which retains more than 99% of EEG power envelope variance.)

A HMM with *K* = 6 states (Quinn et al., 2018) was then inferred from the *N* principal component time courses using variational Bayesian optimization, under several assumptions such as the normality of the observation model or the prior that hidden model parameters follow conjugate distributions (making a parametric optimization possible; for further details, see ((Rabiner, 1989; Rezek and Roberts, 2005)). Of note, the low dimensionality for EEG (*N* = 10, presumably due to high spatial smoothness of EEG; see, e.g., (Coquelet et al., 2020a)) was still sufficient to infer *K* = 6 states. The HMM optimization algorithm was run ten times, each with different initial conditions, and the model with lowest free energy was retained (Baker et al., 2014). Binary time series of most probable, temporally exclusive, and complete state activation were then derived using the Viterbi algorithm (Rezek and Roberts, 2005).

Importantly, the mere difference in number of states for the HMM and microstate analyses may trivially induce discrepancies between them. We controlled for this possibility by re-running the HMM inference with *K* = 4 (see supplementary material S6) and the microstate classification with *K* = 6 (see supplementary material S7).

### 2.6 State temporal properties and power maps

State activation time series allowed to compute several summary statistics of the temporal behavior of microstates or HMM states, such as their mean lifetime (mean duration of state activation events) and their fractional occupancy (fraction of the total recording time during which the state is active). The global effects of recording modality and state clustering algorithm on these statistics were assessed using two-sided paired Student's *t* tests at p<0.05 applied to their average across the *K* states.

Activation time series also allowed to produce spatial maps locating where in the brain power increases or decreases occur upon state activation. Such brain power maps were built as images of the partial correlation between each state activation time series and each MNE source power envelope signal concatenated across subjects (Baker et al., 2014). Corresponding maps could also be derived at the individual level by mere restriction of these partial correlations within each subject. In the sensor-level HMM, this procedure was also performed at the level of sensor power envelope signals used for state inference.

All these maps were thresholded statistically using two-tailed parametric correlation tests at p<0.05 against the null hypothesis that Fisher-transformed correlations follow a Gaussian with mean zero and SD $\frac{1}{\sqrt{\nu -3}}$, where $\nu =N_{tdof}-\left(K-1\right)$. The number *N_tdof_* of temporal degrees of freedom was estimated as one-quarter of the total number of time samples in group-concatenated envelope signals at 40 Hz sampling frequency to take into account the low-pass filter at 10 Hz. The subtraction of *K* – 1 degrees of freedom is due to the regression inherent to the partial correlation. The critical *p*-value was Bonferroni corrected with the number of independent states (i.e., *K* – 1) multiplied by the number of spatial degrees of freedom estimated from the rank of the forward model (Wens et al., 2015), i.e., 58 for MEG and 32 for EEG. Statistical thresholding on state power maps was thus slightly tighter for MEG than EEG, which is merely a reflection of the higher spatial smoothness in EEG data (Coquelet et al., 2020a).

### 2.7 State correlation analyses

The spatial and temporal profiles of each pair of states were compared quantitatively using correlation analyses. The spatial similarity of two states was assessed using Pearson correlation of their source-level brain power maps, and their tendency to co-activate using Spearman correlation of their binary activation time series, both computed within each subject. Statistical significance was then established using one-sided one-sample parametric *t*-tests against the null hypothesis that the group-averaged sample correlation vanishes (reflecting the absence of topographical resemblance or of temporal co-activation between two states) and with the alternative hypothesis that this average is positive (reflecting significant topographical overlap or temporal co-activation). The significance level was set to p<0.05 Bonferroni corrected for the number of possible state pairs included in the comparison at stake.

### 2.8 Data and code availability statement

The MEG/EEG data and analysis code used in this study will be made available upon reasonable request to the corresponding author and after approval of institutional authorities (CUB Hôpital Erasme and Université libre de Bruxelles).

## 3 Results

### 3.1 Direct comparison of EEG microstates and hidden Markov modeling of MEG source power envelopes

**Fig. 1:**
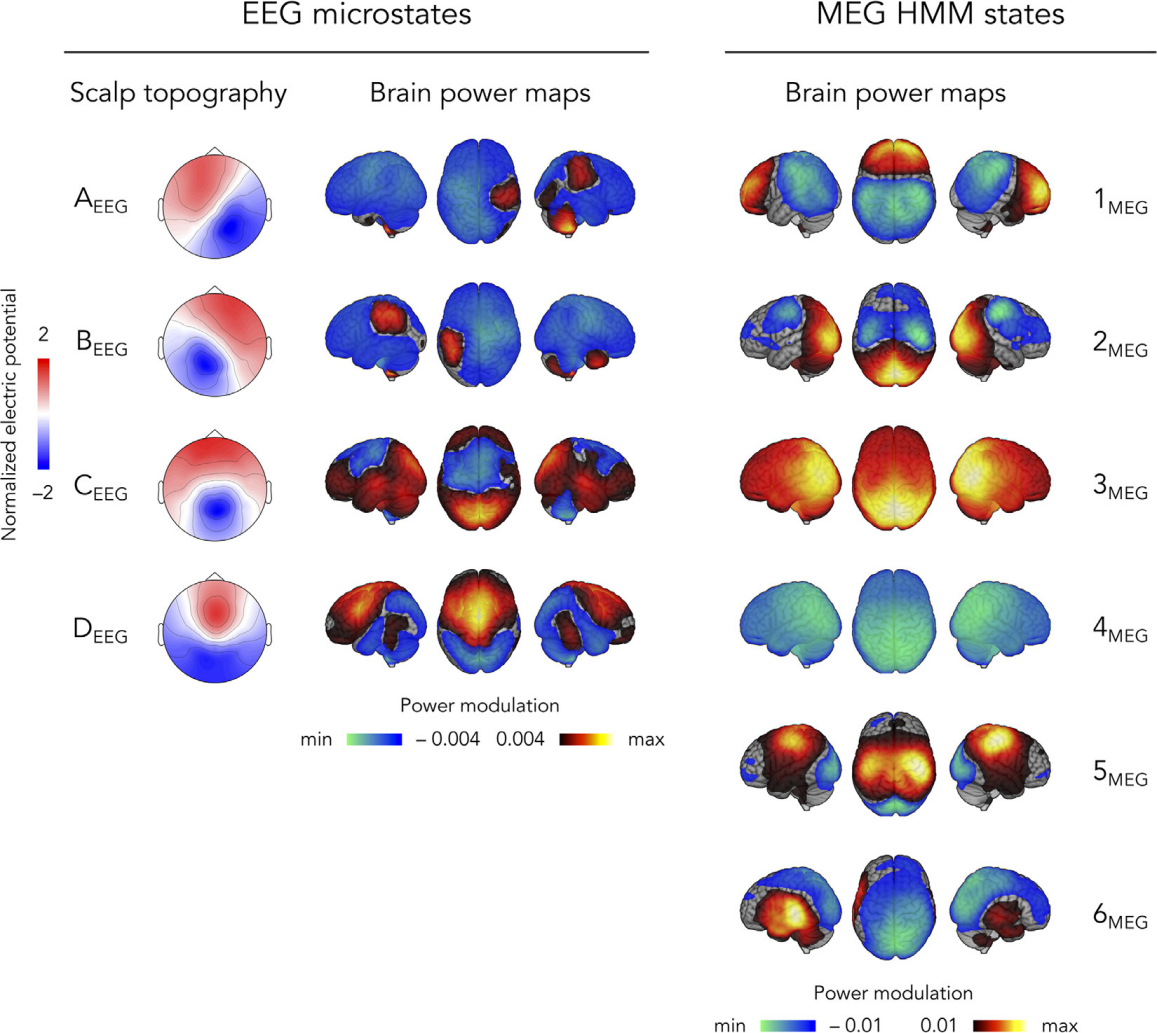
Direct comparison of microstates (**left**) and power envelope HMM states (**right**). The scalp topographies of EEG microstates (four-cluster AAHC of the 200 Hz-downsampled sensor maps at time points of local GFP maxima) are shown after normalization with respect to their GFP and alongside their associated source-level brain power maps. Different color scales are used to emphasize their difference. The HMM states of source-projected MEG power envelopes are visualized as brain power maps as well. Positive (negative) values in the brain power maps indicate increasing (decreasing) power upon state activation. The scale of these brain power maps represents partial correlation values which were thresholded statistically, and the lower/upper limits are adapted to the minimum/maximum values. Note that the statistical thresholding of brain power maps is slightly tighter for MEG HMM than for EEG microstates due to a difference in the number of temporal degrees of freedom.

We started with a comparison of the spatial signature and temporal dynamics disclosed by microstates as classically obtained from EEG signals (AAHC with *K* = 4 applied to 200 Hz-downsampled sensor maps at time points of local GFP maxima, with temporally smoothed microstate activation time courses) on the one hand, and MEG power envelope HMM (*K* = 6 states inferred from 40 Hz-downsampled MNE source power envelopes) on the other hand. The scalp topographies of the four microstates are shown in Fig. 1 (left) alongside the corresponding brain power maps that locate significant power increases (positive values) and decreases (negative values) upon microstate activation. Microstates were sorted and labeled so as to match the denomination typically used in the literature (see, e.g., (Michel and Koenig, 2018)), and scalp topographies were normalized with respect to their GFP. The brain power maps of the six HMM states are depicted in Fig. 1 (left), in no particular order. These maps locate significant power increases/decreases upon HMM state activation.

#### 3.1.1 Microstates

Fig. 1 (left) reproduces the canonical scalp topographies well established in the literature (Michel and Koenig, 2018). Each microstate displayed a scalp potential distribution reminiscent of one current dipole (notwithstanding the difficulty of interpreting scalp EEG in this way; see, e.g., (Hari and Puce, 2017)) approximately located centrally and oriented along the right posterior to left frontal line (microstate A_EEG_), the left posterior to right frontal line (microstates B_EEG_), the posterior to anterior midline (microstate C_EEG_), or predominantly vertically (microstate D_EEG_). The corresponding brain power maps in Fig. 1 (left) allowed to identify cortical areas modulated by microstate activation. Microstate A_EEG_ was dominated by a broad power decrease peaking at the left sensorimotor cortex alongside a weaker power increase at the right sensorimotor cortex. Microstate B_EEG_ exhibited an opposite pattern. Microstate C_EEG_ was dominated by a power increase at the visual occipital cortex, alongside a weaker power decrease peaking in the midline frontal area, and microstate D_EEG_ involved an opposite pattern. All four brain power maps also disclosed deep cerebellar patterns that may be related to EEG source reconstruction errors.

#### 3.1.2 Hidden Markov model states

In accordance with previous studies (Baker et al., 2014; Brookes et al., 2018; Coquelet et al., 2020b), the HMM states identified MEG power modulations within well-known intrinsic functional networks, here the bilateral sensorimotor network (SMN), the visual occipital network (VoN), the posterior part of the default-mode network (pDMN; encompassing the precuneus), and a presumed bilateral auditory network (AN). More specifically, state 1_MEG_ displayed a pattern of SMN deactivation along with pre-frontal activation. State 2_MEG_ involved SMN deactivation alongside pDMN/VoN activation, and state 5_MEG_ showed an opposite pattern, i.e., SMN power activation along with pDMN/VoN power deactivation. These two states are thus reminiscent of a dynamic competition between the SMN and pDMN/VoN (Wens et al., 2019). States 3_MEG_ and 4_MEG_ identified pDMN activation and deactivation, respectively. Of note, similar states involving precuneus activity were also identified in previous works (Coquelet et al., 2020b; Puttaert et al., 2020) and not in others (e.g., (Baker et al., 2014; Brookes et al., 2018)) due to different choices of source projection (for details, see (Sjøgård et al., 2019)). Finally, state 6_MEG_ involved AN activation alongside deactivation at the precuneus, which is once again reminiscent of a dynamic cross-network competition (Wens et al., 2019).

#### 3.1.3 State correlations

**Fig. 2:**
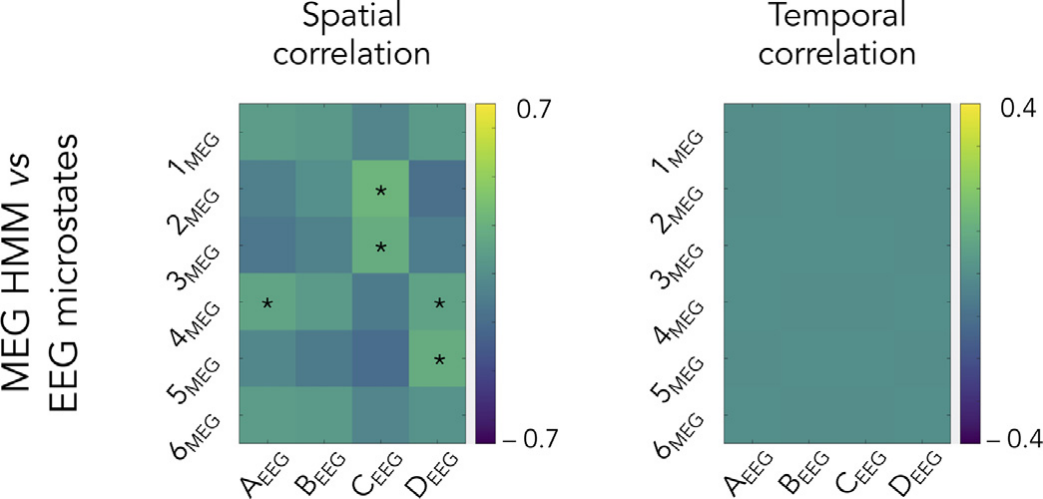
Spatial (**left**) and temporal (**right**) correlations between EEG microstates (four-cluster AAHC of the 200 Hz-downsampled sensor maps at time points of local GFP maxima) and six-state HMM of source-projected MEG power envelopes. Spatial correlations were estimated between brain power maps and temporal correlations, between temporally smoothed microstate activation time series and HMM state activation time series upsampled to 200 Hz. The correlation scales match those of Fig. 5. Stars denote significant correlations after Bonferroni correction for the number of state pairs involved in each comparison.

Fig. 2 shows the group-level spatial and temporal correlations between EEG microstates and MEG power envelope HMM states, the former to quantify the spatial correspondence of their brain power maps discussed qualitatively above and the latter, their tendency to co-activate. Given the different sampling rates for microstates (200 Hz) and HMM states (40 Hz), temporal correlations required upsampling HMM state activation time courses to 200 Hz beforehand.

A number of significant spatial correlations emerged between three microstates and four HMM states (Fig. 2, left; significant $R>0.11,\;t_{41}>3.2,\;p<0.0013\;$ Bonferroni corrected for 24 comparisons). Inspection of the corresponding brain power maps (Fig. 1) revealed that most of these correlations merely reflect the overlapping involvement of brain areas close to the visual occipital cortex (VoN activation for microstate C_EEG_ vs. pDMN/VoN activations for HMM states 2_MEG_ and 3_MEG_; VoN deactivation for microstate D_EEG_ vs. pDMN/VoN deactivations for HMM states 4_MEG_ and 5_MEG_). The correlation between microstate A_EEG_ and HMM state 4_MEG_ appears to be an artefact of MEG/EEG spatial smoothness, which led to a large area of power deactivation in both maps.

On the other hand, temporal correlations were not significant and actually exhibited very small effect sizes ($R<0.003,\;t_{41}<1.58,\;p>0.06\;$ uncorrected). This demonstrates that microstates and HMM states barely co-activate. This result further suggests that the above-mentioned spatial correlations do not reflect a state-specific relationship, and points at a lack of correspondence between EEG microstates and HMM states of MEG source power envelopes.

### 3.2 Effects of state clustering and recording modality

We sought to untangle the factors underlying the discrepancy between EEG microstates and MEG power envelope HMM states by comparing specifically the results of microstate and HMM state classification algorithms applied on MEG/EEG signals processed with the same filters.

#### 3.2.1 Microstates

**Fig. 3:**
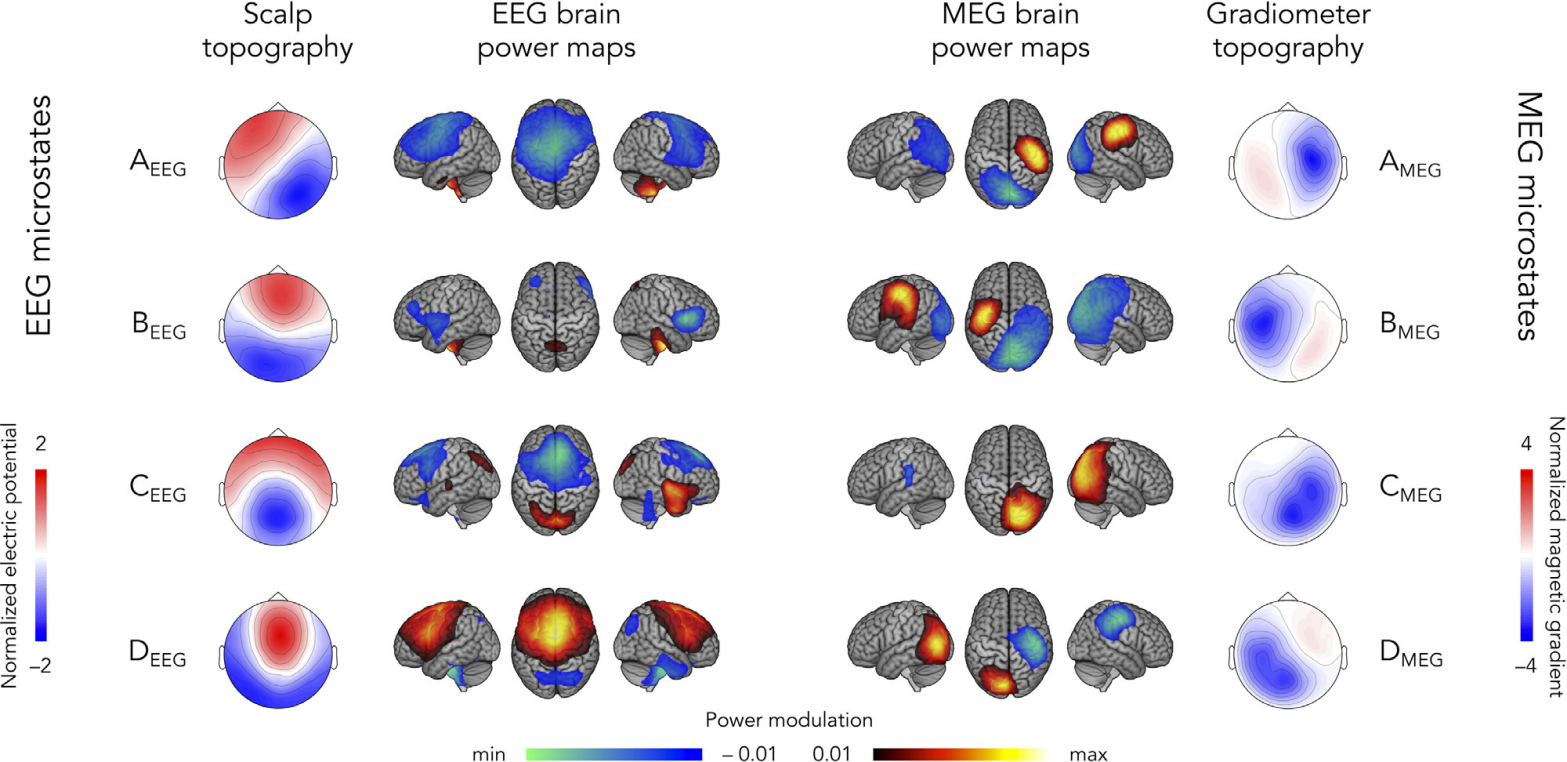
Spatial signature of EEG (**left**) and MEG (**right**) microstates. The scalp topography of EEG microstates (four-cluster AAHC of the 40 Hz-downsampled sensor maps at time points of local GFP maxima) is shown on the far left and the corresponding brain power maps on the middle left. The gradiometer topography of MEG microstates is shown on the far right and the corresponding brain power maps on the middle right. Scales for sensor-level topographical maps and source-level brain power maps are shown using different colors to emphasize their difference. The scales for sensor topographies correspond to electric potential (EEG) or magnetic gradient (MEG) distributions of each microstate normalized to their GFP. Positive (negative) values in the brain power maps indicate increasing (decreasing) power upon microstate activation. The scale of these brain power maps represents partial correlation values which were thresholded statistically, and the lower/upper limits are adapted to the minimum/maximum values.

For definiteness and comparability with the HMM approach, we focus on sensor-level MEG/EEG data downsampled at 40 Hz. Fig. 3 shows the spatial signature of the four microstates derived from EEG and MEG resting-state data (AAHC with *K* = 4 applied to 40 Hz-downsampled sensor maps at time points of local GFP maxima). The EEG microstates (Fig. 3, left) were sorted and labeled according to Fig. 1. For MEG (Fig. 3, right), microstate labels were arbitrary and no pairing with EEG microstates was attempted given the lack of spatial comparability between electric potentials (EEG) and magnetic field gradients (MEG gradiometers) (Hari and Puce, 2017). In both cases, sensor-level topographical maps were normalized with respect to their GFP. The spatial distribution of microstates can be compared across recording modalities only based on the source-level brain power maps that identify power increases/decreases upon microstate activation.

The EEG microstate scalp topographies in Fig. 3 (left) exhibit a high correspondence with canonical microstates (Michel and Koenig, 2018) and those obtained from 200-Hz sampling rate EEG signals (Fig. 1, left). In fact, the lowest spatial correlation of scalp topographies between those of Fig. 1 (left) and Fig. 3 (left) was R=0.83 (corresponding to microstates B_EEG_)_._ This indicates that changing the sampling rate from 200 Hz to 40 Hz has no substantial effect on the spatial signature of microstates. The main noticeable difference is that power modulations in the corresponding brain power maps appear narrower at 40 Hz sampling rate, but this is a mere consequence of the reduction in the number $N_{tdof}$ of temporal degrees of freedom that increases the statistical threshold.

We further examined temporal characteristics of microstates such as their mean lifetime to assess the impact of different options regarding signal filtering and temporal smoothing of microstate activation time courses. Although the spatial signature of these microstates matches the literature, it turns out that their temporal statistics differed substantially, with mean lifetimes shorter than expected (mean ± SD: 37 ± 2 ms, range: 35–38 ms; see Table 1, left). These lifetimes were only moderately longer (mean ± SD: 57 ± 6 ms; range: 55–58 ms) when widening the signal frequency band (1–40 Hz; see supplementary material S1) and even shorter (mean ± SD: 14 ± 1 ms, range: 13–15 ms) when clustering EEG topographies at a higher sampling rate of 200 Hz or when increasing the number of microstates to *K* = 6 (see supplementary material S7). The cause of this discrepancy appeared to be the absence of temporal smoothing on the microstate activation time series in this implementation, as the interpolation approach allowed recovery of typical lifetimes (mean ± SD: 126 ± 8 ms, range: 121–138 ms; see also supplementary material S4). Fractional occupancies ranged from 19% to 27% (Table 1, left) and were not substantially affected by temporal smoothing (23–28%).

**Table 1:** Mean lifetimes and fractional occupancies (mean ± SD) associated with each microstate inferred from EEG or MEG topographies at 40 Hz sampling rate and without temporal smoothing on microstate activation time series.

EEG microstates			MEG microstates		
	Mean lifetimes (ms)	Fractional occupancies (%)		Mean lifetimes (ms)	Fractional occupancies (%)
A_EEG_	38 ± 3	26.9 ± 4.4	A_MEG_	32 ± 1	14.7 ± 1.8
B_EEG_	35 ± 2	19.5 ± 3.9	B_MEG_	37 ± 2	25.1 ± 3.7
C_EEG_	37 ± 3	25.8 ± 4.8	C_MEG_	47 ± 2	41.1 ± 2.9
D_EEG_	38 ± 2	27.6 ± 4.3	D_MEG_	34 ± 2	19.1 ± 2.3

A similar analysis applied to MEG gradiometer signals led to microstates that were dominated by dipolar sensor topographies (Fig. 3, right), much like EEG microstates. Microstates A_MEG_ and B_MEG_ were characterized by gradiometers peaking respectively above the right and the left parietal sensors, which was explained by unilateral power increases at the sensorimotor cortices. These two microstates also involved an occipital power decrease. Microstates C_MEG_ and D_MEG_ disclosed respectively right and left parieto-occipital gradiometer activity corresponding to unilateral occipital power increases. The brain power map for microstate D_MEG_ also showed right sensorimotor power decrease. The neural generators behind MEG microstates thus appeared qualitatively different from those of EEG microstates.

On the other hand, their mean lifetimes (mean ± SD: 37 ± 7 ms, range: 32–47 ms; Table 1, right) tended to be similar ($t_{41}=1.8,\;p\;=\;0.08$). Fractional occupancies appeared less homogenous for MEG (14–41%; Table 1, right) than for EEG, with microstate C_MEG_ showing the highest fractional occupancy. Analogously to the EEG case, temporal smoothing on the MEG microstate activation time series lengthened lifetimes substantially (mean ± SD: 114 ± 21 ms, range: 94–141 ms; see supplementary material S4) but did not affect fractional occupancies (17–38%). Widening the frequency band lengthened lifetimes moderately (mean ± SD: 55 ± 4 ms; range : 37 – 102 ms; see supplementary material S1) and increasing the sampling rate further shortened lifetimes (mean ± SD: 11 ± 3 ms, range: 8–16 ms). It is noteworthy that these lifetimes were also not influenced by the MEG system type (unpaired *t*-tests between the group of 15 participants scanned with the Vectorview system and the group of 27 participants scanned with the Triux system, $|t_{40}|<1.95,\;p\;>0.24$ Bonferroni corrected for *K* = 4 microstates).

It is finally interesting to mention that the fraction of global variance explained by the microstate classification (Murray et al., 2008) was larger for EEG (55%) than MEG (35%), which is reminiscent of the fact that EEG data are spatially smoother, and thus of lower dimensionality *N*, than MEG data (as was explained above). The fraction of global explained variance increased slightly (61% in EEG and 37% in MEG) when increasing the number of microstates to *K* = 6 (see supplementary material S7).

Several minor variations of the microstate clustering approach are explored in supplementary materials. These analyses show that the above features of microstates are robust against methodological changes such as widening the signal frequency band (except for the effect on lifetimes, see supplementary material S1), using group clustering (supplementary material S2), or lifting the restriction to GFP local maxima (supplementary material S3). Further, increasing the number of microstates to *K* = 6 preserved the four microstates identified for *K* = 4 and did not substantially modify the clustering model, especially for MEG (see supplementary material S7 for details).

#### 3.2.2 Hidden Markov model states

**Fig. 4:**
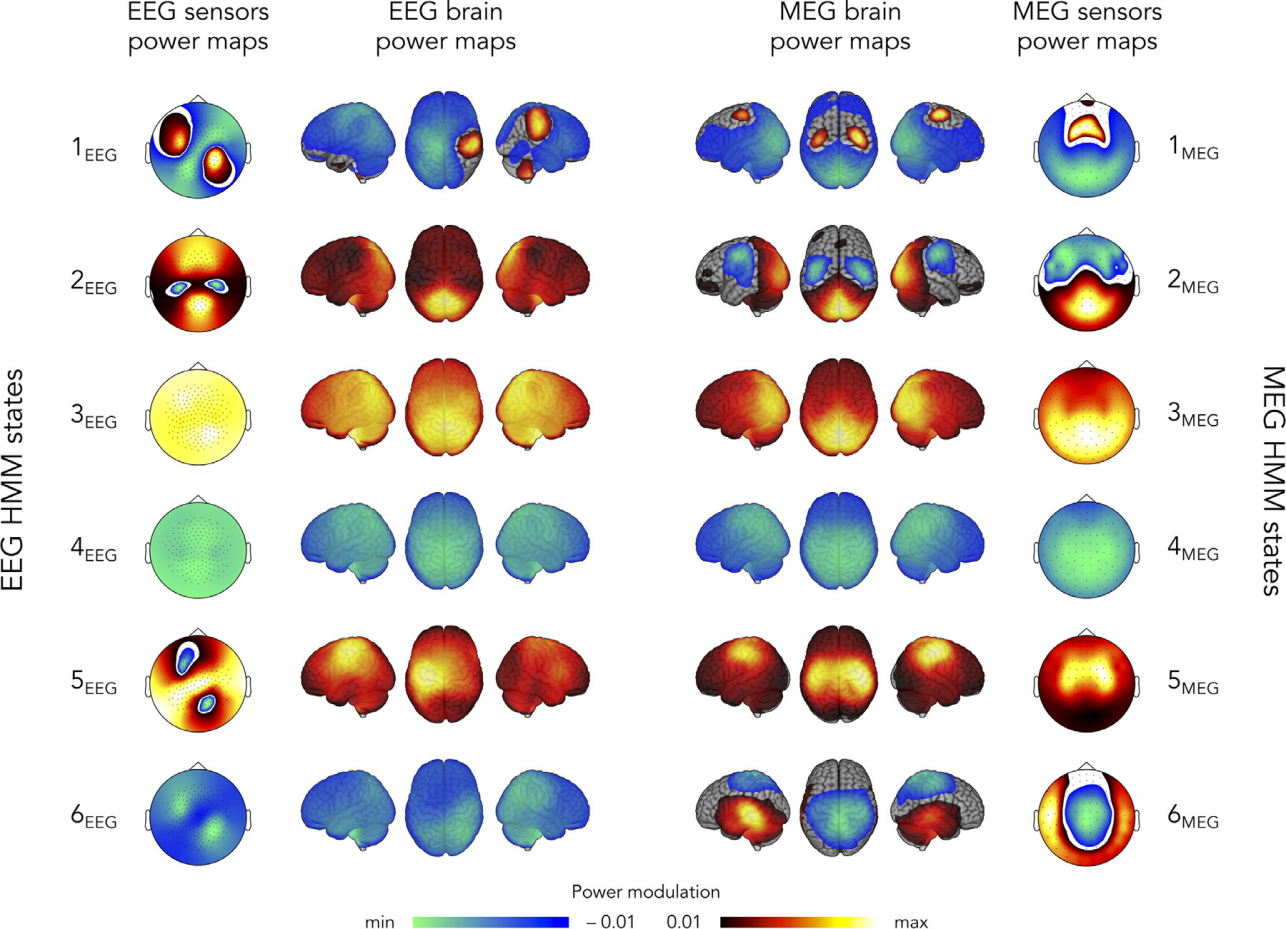
Spatial signature of EEG (**left**) and MEG (**right**) sensor-level power envelope HMM states. Both sensor and brain power maps locate power increases (positive values) and decreases (negative values) upon state activation. The scale of these power maps represents partial correlation values which were thresholded statistically, and the lower/upper scales are adapted to the minimum/maximum values. States were ordered and labelled based on a visual pairing of EEG and MEG brain power maps.

For comparability with the microstate clustering based on sensor signals, we focus on a version of the HMM analysis based on the power envelopes of resting-state MEG/EEG sensor data rather than source-projected data. Fig. 4 depicts the spatial signature of the resulting six HMM states, which were sorted and labeled in order to pair EEG states (Fig. 4, left) and MEG states (Fig. 4, right) with the best apparent spatial correspondence in their brain power maps. An important difference with Fig. 3 is that the sensor maps in Fig. 4 do not disclose electric potential or magnetic field gradient topographies (as was the case in Fig. 3), but rather sensor-level power increases or decreases upon state activation, in complete analogy with the brain power maps that locate source-level power modulations. Both sensor and brain power maps are directly comparable for MEG because planar gradiometers are sensitive to source activity just beneath them, but this comparison is less straightforward for EEG given the source sensitivity profile of electrodes (Hari and Puce, 2017). For this reason, we focus in the following on a description of their brain power maps.

We start with the MEG states (Fig. 4, right). Comparison with Fig. 1 (left) shows that MEG power envelope HMM inference based on sensor signals and source signals led to brain power maps that are largely similar. The only two qualitative differences were that power activation was located in the SMN rather than in prefrontal areas for state 1_MEG_, and that the SMN was activated in isolation rather than in competition with the pDMN/VoN for state 5_MEG_. We conclude that there is a good spatial correspondence between power envelope HMM states inferred from sensor and source-projected MEG signals.

State mean lifetimes obtained with this sensor-level HMM (mean ± SD: 151 ± 31 ms; range: 128–211 ms; see Table 2, right) were significantly shorter than with the source-level HMM ($t_{41}=5.1,\;p\;=\;2.7\times 10^{-6}$), but still significantly longer than the mean lifetime of non-smoothed MEG microstates, i.e., without temporal smoothing of microstate activation time series ($t_{41}=31.6,\;p\;=\;0$). Fractional occupancies ranged between 7% and 23% (Table 2, right). Similarly to the MEG microstates, the lifetime of MEG power envelope HMM states was not significantly impacted by the type of MEG recording system ($|t_{40}|\leq 2.39,\;p\;\geq 0.12$ Bonferroni corrected for *K* = 6 states).

**Table 2:** Mean lifetimes and fractional occupancies (mean ± SD) associated with each of the six HMM states inferred from EEG or MEG power envelope signals.

EEG HMM			MEG HMM		
	Mean lifetimes (ms)	Fractional occupancies (%)		Mean lifetimes (ms)	Fractional occupancies (%)
State 1_EEG_	136 ± 19	17 ± 3.7	State 1_MEG_	138 ± 34	22.2 ± 10.9
State 2_EEG_	145 ± 39	13.3 ± 4.5	State 2_MEG_	144 ± 41	16.4 ± 8.2
State 3_EEG_	204 ± 70	8.4 ± 1.4	State 3_MEG_	153 ± 52	7.5 ± 3.2
State 4_EEG_	226 ± 109	22.4 ± 9	State 4_MEG_	211 ± 98	23.1 ± 10.6
State 5_EEG_	137 ± 20	14.6 ± 4.7	State 5_MEG_	128 ± 40	12.5 ± 7.7
State 6_EEG_	141 ± 23	24.3 ± 6.2	State 6_MEG_	130 ± 54	18.3 ± 15.4

The power envelope HMM states inferred from scalp EEG involved power modulations within intrinsic networks similar to the MEG states, although not with the same degree of bilaterality (Fig. 4, left). State 1_EEG_ was characterized by the activation of the right part of the SMN alongside a power decrease in the left precuneus, and as such may be viewed as a unilateral version of MEG state 1_MEG_. This state was the only EEG state exhibiting both power increases and decreases. The VoN activation state 2_EEG_ was comparable to state 2_MEG_ but lacked SMN deactivation, and the pDMN states 3_EEG_ and 4_EEG_ closely matched states 3_MEG_ and 4_MEG_. State 5_EEG_ was characterized by a power increase in the left part of the SMN, so it appeared as a unilateral version of state 5_MEG_. Finally, state 6_EEG_ consisted in a right-hemispheric posterior parietal power decrease, which was thus qualitatively different from the AN/precuneus state 6_MEG_. The mean lifetime of these states (mean ± SD: 165 ± 40 ms, range: 136–204 ms; see Table 2, left) was significantly longer than the non-smoothed EEG microstates ($t_{41}=31.46,\;p\;=\;0$) and the MEG HMM states ($t_{41}=4.83,\;p\;=\;1.9\times 10^{-5}$). Fractional occupancies were between 8% and 24%, which is also similar to those observed using MEG (Table 2).

It is noteworthy that increasing the dimensionality of the EEG data inputted to the HMM algorithm to the same dimension used in MEG, led to qualitatively similar states, although with a higher degree of bilaterality for some states (supplementary material S5). Reducing the number of states to four led to HMM states closely related to states 2_MEG_–5_MEG_ for MEG and to states 1_EEG_, 3_EEG_–5_EEG_ for EEG (supplementary material S6).

#### 3.2.3 State correlations

**Fig. 5:**
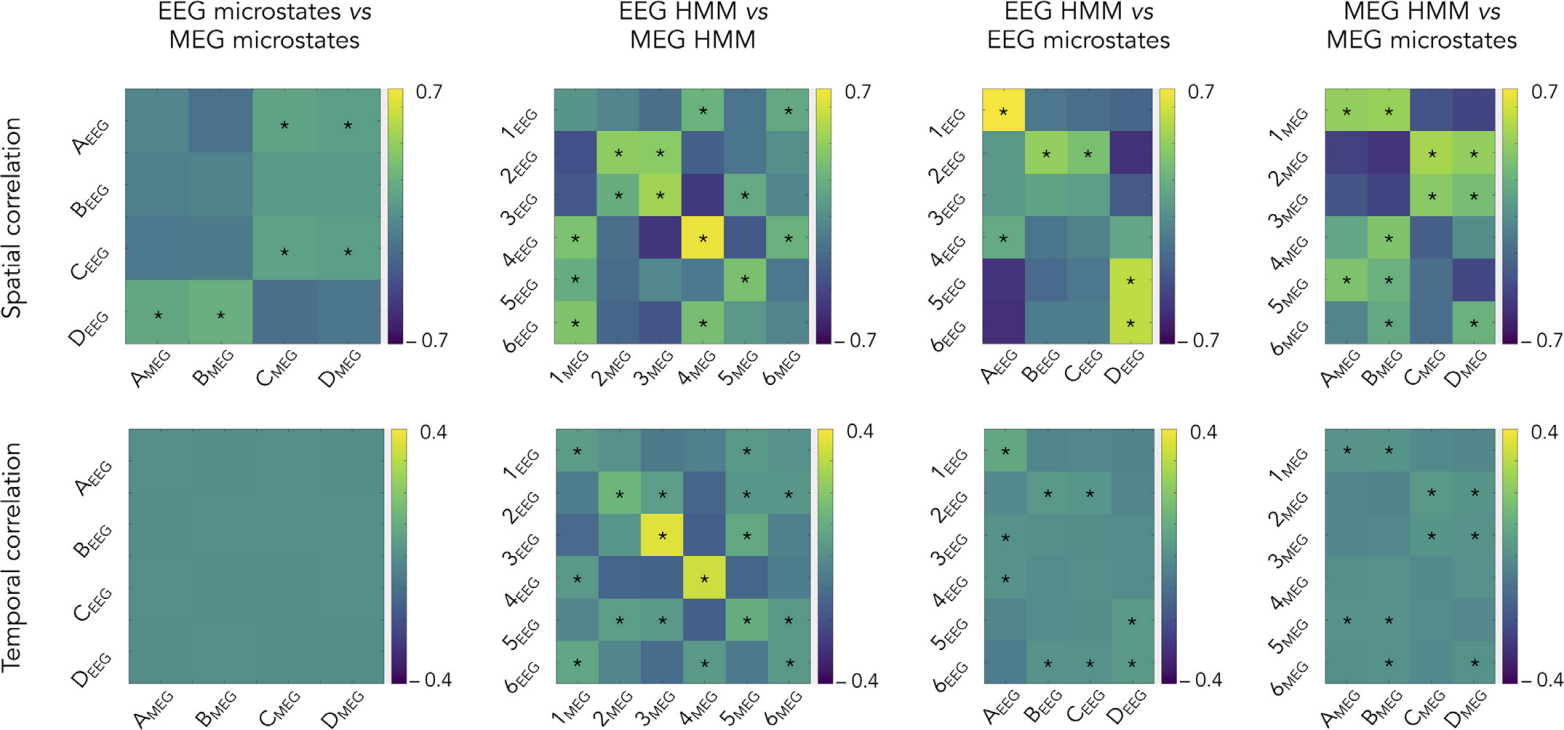
Spatial (**top**) and temporal (**bottom**) state correlations. Each matrix shows the group-level correlation values comparing: EEG microstates vs. MEG microstates (**first column**; corresponding to four-cluster AAHC of the 40 Hz-downsampled sensor maps at time points of local GFP maxima), EEG HMM states vs. MEG HMM states (**second column**; six-state HMM of sensor-level power envelopes), EEG HMM states vs. EEG microstates (**third column**), and MEG HMM states vs. MEG microstates (**fourth column**). Temporal correlations were obtained from the raw (non-smoothed) microstate activation time series. The same correlation scale is used across the four comparisons. Stars denote significant correlations after Bonferroni correction for the number of state pairs involved in each comparison.

The spatial correspondence among different states was assessed statistically using spatial correlations of brain power maps (Fig. 5, top). Brain power maps were used rather than sensor topographies given their lack of comparability across recording modalities. The degree of state co-activations was estimated in terms of temporal correlations of state activation time courses (Fig. 5, bottom). We detail results obtained using non-smoothed microstate activation time series, but it is noteworthy that temporal smoothing yielded very similar results (see supplementary material S4 for details). The two first columns of Fig. 5 assess the effect of recording modality (MEG vs. EEG) on microstates (Fig. 5, first column) and HMM states (Fig. 5, second column) and the two last columns, the effect of state clustering algorithm (microstate vs. HMM) for both EEG (Fig. 5, third column) and MEG (Fig. 5, fourth column). It is worth mentioning that both spatial and temporal correlations between MEG microstates and MEG power envelope HMM states were not significantly affected by the MEG system type ($|t_{40}|\leq 2.66,\;p\;\geq 0.53$ Bonferroni corrected for two types of correlation matrices and 24 entries each).

The comparison of EEG vs. MEG microstates confirmed the absence of a clear relationship. Some cross-modal pairs did disclose significant spatial correlations (Fig. 5, top of left column; significant $R>0.09,\;t_{41}>3.21,\;p<0.021\;$ Bonferroni corrected for 16 comparisons). They could be explained by a gross overlap of their power maps presumably due to their intrinsic blurriness, i.e., EEG microstates A_EEG_ and C_EEG_ tended to exhibit posterior power increases and antero-central power decreases as did the MEG microstates C_MEG_ and D_MEG_, and reversely for microstates D_EEG_, A_MEG_, and B_MEG_ (Fig. 3). More importantly, the corresponding temporal correlations were not significant with very small effect sizes (Fig. 5, bottom of left column; $R<0.002,\;t_{41}<1.14,\;p>0.13\;$ uncorrected), indicating that EEG and MEG microstates scarcely co-activate at all.

On the other hand, Fig. 5 (second column) revealed a number of significant correlations between MEG and EEG HMM states, both spatially (significant $R>0.15,\;t_{41}>3.85,\;p<7.2\times 10^{-3}\;$ Bonferroni corrected for 36 comparisons) and temporally (significant $R>0.025,\;t_{41}>4.02,\;p<4.2\times 10^{-3}\;$ corrected), and with higher effect sizes and smaller *p* values than microstates. The qualitative pairing of MEG and EEG states based on their brain power maps (Fig. 4) was reflected in the significance along the diagonal of the correlation matrix (Fig. 5, top of second column), with particularly high effect size and low *p* value for the pDMN deactivation state 4_MEG_/4_EEG_ ($t_{41}=32.73,\;p=0$). The two exceptions were states 1_MEG_/1_EEG_ (where the correlation did not reach significance presumably due to the sign reversal above the left sensorimotor cortex) and states 6_MEG_ and 6_EEG_. Analogously to the case of microstates, and given the qualitative pairing of MEG and EEG states (Fig. 4), off-diagonal significance may be a reflection of spatial blurriness. Temporal correlations followed a similar pattern (Fig. 5, bottom of second column), with the pDMN states 3_MEG_/3_EEG_ and 4_MEG_/4_EEG_ standing out regarding their effect size and *p* value ($t_{41}>14.11,\;p=0$). Globally, the HMM inference on power envelopes was thus able to identify common states across the two recording modalities.

We turn now to the comparison of microstates and HMM states within each modality. Spatial correlations appeared significant in a number of microstate/HMM state pairs (EEG: Fig. 5, top of third column, significant $R>0.16,\;t_{41}>3.2,\;p<0.031\;$ Bonferroni corrected for 24 comparisons; MEG: Fig. 5, top of fourth column, significant $R>0.15,\;t_{41}>4.02,\;p<2.29\times 10^{-3}\;$ corrected). For EEG, these correlations mainly reflect spatial blurriness since microstate and HMM states power maps peaked at distinct locations, except for similar power increases at the visual cortices (microstates B_EEG_, C_EEG_ and HMM state 2_EEG_, see Figs. 3 and 4, left). This is important since spatial extent is not interpretable in EEG/MEG source reconstructed maps, but peak localization is (Bourguignon et al., 2018). For example, the strongest spatial correlation emerged between HMM state 1_EEG_ and microstate A_EEG_, however the former exhibits a power decrease located at the left parietal cortex (Fig. 4) and the latter, a power decrease at the midline posterior frontal area (Fig. 3). This correlation is thus driven by the large blurr of negative values in both maps. For MEG, Figs. 4 and 5 (right) indicated some degree of co-localization in microstate and HMM state power modulation within the SMN (activation for microstates A_MEG_, B_MEG_ and HMM states 1_MEG_, 5_MEG_; deactivation for microstate D_MEG_ and HMM state 2_MEG_) and VoN (activation for microstates C_MEG_, D_MEG_ and HMM states 2_MEG_, 3_MEG_). Temporal correlations followed once again a somewhat similar pattern of significance (EEG: Fig. 5, bottom of third column, significant $R>0.01,\;t_{41}>3.19,\;p<0.034\;$ corrected; MEG: Fig. 5, bottom of fourth column, significant $R>0.007,\;t_{41}>3.52,\;p<0.013\;$ corrected). However, the raw value of these significant correlations remained low (EEG: R<0.09, MEG: R<0.04), so temporal co-activations were marginal. This could merely reflect the fact that both clustering methods were applied on the same signals.

Importantly, equalizing the number of HMM states and of microstates (by decreasing the number of HMM states to *K* = 4, see supplementary material S6; or by increasing the number of microstates to *K* = 6, see supplementary material S7) did not alter our main observations on the lack of temporal co-activation. In fact, temporal correlations between microstates and HMM states remained marginal. Our results thus appear robust against changes in the number of microstates and HMM states at stake.

## 4 Discussion

This study used simultaneous MEG/EEG recordings at rest to compare two notions of discrete metastable brain states, i.e., microstates and power envelope HMM states. Direct comparison of classical implementations of EEG microstate analysis and source-projected MEG power envelope HMM revealed a poor correspondence between the two types of states. Exploring further the roots of this discrepancy, we found that microstates were not reproducible across the two recording modalities, i.e., microstates inferred from MEG signals did not correspond to the canonical EEG microstates. On the other hand, MEG and EEG HMM states identified transient activations of similar intrinsic functional networks, with a related, but marginal, temporal correspondence. We also found no clear evidence that microstates and HMM states share common neural dynamics, as spatio-temporal correlations appeared sensitive to biases such as the blurriness of source reconstructions. In fact, contrary to our expectation based on the literature (Baker et al., 2014; Michel and Koenig, 2018), all microstates were substantially less stable in time than the HMM states (at least in the absence of *ad-hoc* temporal smoothing of microstate activation). That said, the MEG version of microstates involved power activity within the same networks as HMM states, but was restricted to isolated nodes of these networks, and with a poor temporal correspondence.

### 4.1 Microstates and power envelope HMM states probe different aspects of electrophysiological power bursts

The primary result of this paper is that microstates and power envelope HMM states differ substantially, both in the localization of the brain areas they (de)activate and in their temporal stability. These two state clustering algorithms share the common goal of identifying patterns of high-power electrophysiological activity that repeat at rest, so this raises the questions of what methodological features lead to this discrepancy, and what aspect of brain functional dynamics they are preferentially sensitive to. The fundamental distinction discussed here is that (i) microstates focus on high-power activity by biasing the topographical clustering to time points of locally maximum GFP (Michel and Koenig, 2018), whereas (ii) the power envelope HMM encodes states based on the spatial patterns of continuous-time oscillatory power (Baker et al., 2014).

The GFP maximization for microstate topographies is fully built-in the AAHC algorithm (Murray et al., 2008). In fact, the convergence of the AAHC with and without explicit restriction to GFP peaks indicates that microstates are mostly sensitive to time points of locally maximal GFP. This concurs with the reportedly high levels of EEG topographical dissimilarities in between GFP peaks (Skrandies, 1990) and with the difficulty of discrete microstates to model continuous EEG recordings (Mishra et al., 2020). Accordingly, in our data, the duration of microstate activation appeared very short, and was actually only slightly above the minimum timescale allowed by signal processing (at least in the absence of temporal smoothing). Reaching such small timescales despite the fact that signals were effectively low-pass filtered, was due to the fact that the moving-window averaging technique for downsampling imposes a soft (rather than a hard) filter, allowing higher frequencies to still contribute. Classical lifetimes of 120 ms appear to require an *ad-hoc* temporal interpolation procedure that does not reflect the raw GFP peak events underlying microstate clustering *per se*, nor the high topographical dissimilarities in between these events (Skrandies, 1990). This temporally-smoothed microstate dynamics exhibits by design longer-lived activation events, but these are not necessarily representative of the actual MEG/EEG signal events that underlie the very construction of microstates (i.e., repeating sensor topographies). These events captured by the raw, non-smoothed microstate time series revealed shorter-lived microstate activations with a mean lifetime of 37 ms. This is actually only 150% the 25 ms timestep of our signals sampled at 40 Hz, and the fact that it decreased by merely increasing the sampling rate indicates that microstate events are actually even sharper. Extrapolating the observation that raw microstate lifetimes are 150% the timestep would have led us to expect a mean lifetime of about 7 ms at 200 Hz sampling rate (corresponding to a 5 ms timestep), but our data proved it twice longer. This is presumably a sign that microstates do reflect neural events, since neurophysiological activity as recorded by MEG/EEG should typically not occur over timescales shorter than the 10 ms duration of postsynaptic potentials (Baillet, 2017; Buzsáki et al., 2012), whereas pure noise events can be as short as the timestep. Microstates thus appear to probe quasi-instantaneous electrophysiological events.

Further understanding what these microstate events represent requires careful consideration of the notion of GFP. Instantaneous GFP (spatial variance of time-dependent sensor topographies) is not trivially synonymous with instantaneous global power (magnitude squared signal summed over all sensors). For EEG, the two concepts coincide only when using the average reference (where the potential summed over all electrodes is constrained to vanish), which approaches the idealized reference to infinity because asymptotically vanishing electric potentials generated by current dipoles inside the brain integrate to zero over the scalp (Bertrand et al., 1985), at least to some approximation (Yao, 2017). From a physical perspective, GFP maximization of EEG microstates is thus theoretically equivalent to global power maximization. In practice though, the GFP formulation is preferred because it is strictly independent of the choice of reference (Murray et al., 2008; Skrandies, 1990). No such subtlety arises with MEG, where GFP and global power coincide because neuromagnetic field patterns generated by dipolar brain sources also sum up approximately to zero over whole-head-covering sensor arrays (meaning in a sense that the “average reference” holds automatically for MEG). Thus, the quasi-instantaneous microstate events correspond to moments of high global power. Given that spontaneous electrophysiological activity exhibits power bursts (Hari and Salmelin, 1997; van Ede et al., 2018), microstates may be expected to probe short-time events of maximum power within power bursts. More specifically, since the EEG/MEG spectrum is dominated by the alpha band (Hari and Puce, 2017), microstates are bound to be driven by, and phase-locked to, moments of high-amplitude alpha rhythms within alpha bursts (von Wegner et al., 2021).

In any case, by focusing on quasi-instantaneous and temporally discrete electrophysiological events of high power, microstates provide at best a partial characterization of power bursts. Their full exploration requires instead to focus on the transitioning between low and high power. By running over the whole power envelope signal, the HMM is more sensitive to such transitions and may thus be better suited to fully capture bursting activity (van Ede et al., 2018). The Markovian character of the HMM (i.e., the probability of state activation at the next time step depends on what state is currently active; see, e.g., (Rabiner, 1989)) also enforces a degree of deterministic causality that further helps detecting transient periods of sustained power burst, rather than quasi-instantaneous events of high power. Accordingly, bursts generated by brain rhythms typically last for a few hundreds of milliseconds (Hari and Salmelin, 1997), which is consistent with the typical power envelope HMM mean lifetime of 100–200 ms. The fact that these lifetimes are well above the minimum timestep allowed in our power envelope signals (in our case, 25 ms) further shows that they provide a reliable estimate of the duration of the underlying power bursts.

### 4.2 Microstates identify synchronized neural events whereas power envelope HMM states encompass neural activity across intrinsic networks

Besides temporal stability, microstates and power envelope HMM states also differed in their spatial distribution. Microstates exhibited dipolar scalp topography and power modulations at one isolated region along with a few other subdominant regions. On the other hand, the HMM states disclosed distributed (de)activations of functional networks that are reminiscent of classical resting-state networks as revealed by intrinsic functional connectivity analysis (Baker et al., 2014). The first methodological reason to consider to explain this difference is that microstate clustering relies on topographical similarity (Michel and Koenig, 2018) whereas the HMM encodes the whole covariance structure (Baker et al., 2014; Woolrich et al., 2013). In theory, HMM states are thus driven by a mixture of power topography and intrinsic functional connectivity. This being said, the contribution of functional connectivity (more specifically, the cross-covariance feature in the HMM) may not dominate HMM state inference in practice (Vidaurre et al., 2018). In fact, functional networks can also be identified successfully using classification schemes that do not encode explicitly for envelope cross-covariance, such as the independent component analysis of power envelopes (which is, however, generally applied at slower timescales around 1 s; see, e.g., (Brookes et al., 2011; Wens et al., 2014)).

The involvement of functional networks in HMM states and the lack thereof in microstates might alternatively be rooted in their difference in temporal stability discussed at length above. The physiological process of binding distant neural populations into a functional network entails a hierarchy of timescales, from hundreds of milliseconds accessible to the HMM for certain networks (i.e., SMN, DMN and visual network) to several seconds for others (e.g., the fronto-parietal network) (Baker et al., 2014; Vidaurre et al., 2018). With lifetimes below these timescales (without temporal smoothing) and associated with quasi-instantaneous MEG/EEG events, microstate clustering may thus be mostly sensitive to highly transient neural activity taking place locally without enough time to establish network-level coordination. A somewhat related hypothesis was put forth when comparing EEG microstates to fMRI networks (Britz et al., 2010; Musso et al., 2010; Yuan et al., 2012). This is also in line with our observation that MEG microstates appeared as unilateral versions of some HMM states. For example, correlation results suggested that HMM state 1_MEG_ may be viewed as a combination of microstates A_MEG_ and B_MEG_.

Closely related to this timescale argument, the spatial locality of microstates may also be interpreted in light of their being phase locked to alpha rhythms (von Wegner et al., 2021). A putative “network-level” microstate involving distinct brain regions would then imply the existence of a zero-phase lag synchronization among them, and as such it would presumably not reflect neurophysiological activity. This is because zero-lag synchronization among separated brain areas evidences instantaneous interactions, which are generally thought to be non-physiological (Schoffelen and Gross, 2009; Wens, 2015). That said, the notion of microstate network was put forth by (Custo et al., 2017), who identified brain generators of microstate dynamics mostly in the anterior and posterior midline cortices. The existence of such a (nearly) zero-lag synchronization between these two major regions of the default-mode network is closely related to the identification via MEG functional connectivity of spontaneous linear correlations within that network (Sjøgård et al., 2019). It is however unclear why our microstate brain power maps failed at revealing a similar pattern. One possible reason may be that microstate clustering is sensitive to phase relationships, which are thus reflected in the maps shown in (Custo et al., 2017) and not in our brain power maps as power envelopes ignore phase dynamics. One way to further investigate the relationship between microstates and synchronization would then be to compare them to another implementation of the HMM (Vidaurre et al., 2018) that is not applied on MEG/EEG power envelopes but on the MEG/EEG signals with time-delay embedding (Kantz and Schreiber, 2003; Takens, 1981), which gives access to classification features closely related to phase synchrony (Stam and van Dijk, 2002). Compared to the power envelope HMM, this time-embedded HMM exhibits shorter lifetimes (50–100 ms), richer spectral details, and network-level phase locking (Vidaurre et al., 2018). Given that these lifetimes are still well above the smallest accessible timestep (4 ms at the 250 Hz sampling rate used in (Vidaurre et al., 2018)) and thus cannot be deemed quasi-instantaneous, and that this network synchrony occurred at non-zero phase lag, we surmise that the time-embedded HMM provides yet another state description, more stable than microstates but more transient than power envelope HMM states. Still, it would be useful to perform such comparisons explicitly in the future.

In sum, the above considerations suggest that microstates and HMM states are sensitive to neural events occurring at different timescales, highly transient for the former, more stable and distributed over intrinsic functional networks for the latter.

### 4.3 Cross-modal comparisons reveal poor correspondence of state activations

The conclusion that microstate classification depends on highly transient events is also key to understanding the lack of qualitative correspondence between EEG and MEG microstates. This discordance contrasted with the HMM, which disclosed good spatial similarity across the two recording modalities. The pDMN state pairs 3_MEG_/3_EEG_ and 4_MEG_/4_EEG_ exhibited a substantial overlap of their activation periods, but the others lacked such strong temporal correspondence. The pDMN states were also the most stable (Coquelet et al., 2020b; Puttaert et al., 2020), suggesting that state co-occurrence rate increases with state stability. The difficulty of short-lived states to co-activate explains in particular the poor cross-modal temporal correlation for the quasi-instantaneous microstates. This observation is also in line with a previous comparison of MEG and EEG intrinsic functional connectomes, which were spatially similar but with rather discordant temporal dynamics (Coquelet et al., 2020a). The hypothesis raised to explain this result was that MEG and EEG are sensitive to different components of transient functional integration processes, but that these differences smooth out after minute-scale time averaging. Our results suggest that this smoothing effect extends to the finer timescales accessible to MEG/EEG state analyses. In fact, it fits well with the observation discussed above that some relatively stable, network-level HMM states break into highly transient, spatially local MEG microstates.

The spatial discordance between EEG and MEG microstates can then be understood on this basis. A sensitivity of EEG and MEG to different transient neural events (as hypothesized above) would lead to different GFP maxima and thus, to microstates inferred from totally different time points. More generally, the concept of GFP maxima turns out to be modality specific since it depends on the type of sensors used (here, EEG electrodes vs. MEG gradiometers). We focused in this paper on MEG microstates derived from gradiometers, but it is noteworthy that microstates based on magnetometers also poorly correlate with gradiometric microstates (data not shown). We conclude that microstates obtained with different electrophysiological modalities probe neural events occurring at different times and are thus not directly comparable. As emphasized above, HMM state inference does not depend on a modality-specific selection of time periods, which explains their better cross-modal concordance.

Other potential sources of differences are the distinct sensitivity profiles of EEG and MEG, especially to purely radial dipolar sources (Hari and Puce, 2017), and the higher regional variability of sensor-brain distance with MEG arrays than with scalp EEG (Coquelet et al., 2020a). The latter impacts substantially MEG functional connectivity estimation in frontal regions from which MEG sensors are farthest (Coquelet et al., 2020a), but interestingly no such issue was clearly observable in brain power maps of sub-second HMM states. Still, these differences might partially account for their poor temporal correspondence.

### 4.4 Power envelope HMM states can be inferred directly from sensor-level signals

One side result noteworthy of mention is that the HMM of sensor power signals leads to network-level states similar to the HMM of reconstructed source power considered in the seminal paper of (Baker et al., 2014) and subsequent MEG studies (Brookes et al., 2018; Coquelet et al., 2020b; Puttaert et al., 2020; Quinn et al., 2018; Sitnikova et al., 2018; Van Schependom et al., 2019; Vidaurre et al., 2018). The HMM of electrophysiological signals can thus be performed in a computationally less cumbersome way than previously done, for similar results. This might widen the perspectives of applications of the HMM-based analyses of MEG/EEG data, particularly when studying infants or patients where MRI acquisition might not be possible. Methodologically, this also frees the HMM state inference *per se* from ambiguities related to the choice of forward model (for further discussion of this aspect, see, e.g., (Coquelet et al., 2020a)) and source reconstruction algorithm. Only the imaging of state brain power maps would depend on these choices. This is particularly interesting with regard to the contribution of precuneus activity to HMM state dynamics, as it was identified from MEG source power HMM when using minimum norm estimation (Coquelet et al., 2020b; Puttaert et al., 2020) but not when using a beamformer (Baker et al., 2014; Brookes et al., 2018; Vidaurre et al., 2018) due to a suppression effect (Sjøgård et al., 2019). The states 1_MEG_, 3_MEG_/3_EEG_ and 4_MEG_/4_EEG_ obtained in this study show that sensor-level HMM is sensitive to precuneus activity, independently of source reconstruction biases. This being said, it would be interesting in the future to extend our comparative study to other implementations of the power envelope HMM, e.g., restricted to parcellated source reconstruction and with multivariate signal leakage correction (Brookes et al., 2018; Colclough et al., 2015; Sitnikova et al., 2018), and examine whether such processing steps improve the robustness of HMM state inference.

One last aspect to emphasize in the case of EEG is that, strictly speaking, the power envelope HMM is ill-defined because it relies on the concept of scalp EEG signal power, which depends on the choice of reference. As discussed above, we focused here on the average reference, which approximates the physically ideal reference at infinity and thus presumably mitigates this issue in practice. This is in line with the fact that source-projected brain power maps of sensor-level HMM states correspond to maps of source-level HMM states (data not shown), the latter being based on current dipole estimates that are independent of the reference (of course, the choice of recording reference does matter, as it impacts measurement quality (Hari and Puce, 2017)). Still, sensor-level HMM state inference may be improved by using, e.g., the reference electrode standardization technique that aims at simulating a virtual reference at infinity (Yao, 2001).

## Conclusion

This study revealed that microstates and HMM states reflect neural dynamical events probing power bursts at different timescales. The quasi-instantaneity of microstates explains their specificity to the electrophysiological recording modality at hand. For EEG, microstate analysis and the power envelope HMM appear to bring complementary information about transient neural dynamics, so we suggest that the two approaches should be considered together. On the other hand, the added value of MEG microstates may be more limited as they merely identify a short-time splitting of network-level HMM states. Both approaches allow to model fast, spontaneous bursts of electrophysiological activity occurring at sub-second timescales. As such, they represent important tools to further explore the dynamical functional architecture of the human brain.

## Author contributions

N.C., X.D.T. and V.W. designed study; N.C., L.R., X.D.T and V.W. acquired data; N.C. and V.W. contributed to analysis tools; N.C., X.D.T. and V.W. analysed data; N.C., X.D.T., L.R., P.P., S.G., M.W. and V.W. wrote and reviewed the manuscript.

## Additional information

The authors declare that they have no competing financial interests.

## Availability statement

The MEG/EEG data and analysis code used in this study will be made available upon reasonable request to the corresponding author and after approval of institutional authorities (CUB Hôpital Erasme and Université libre de Bruxelles).

## Appendix Supplementary materials

Image, application 1
