# Supplementary material for: Microstates and power envelope hidden Markov modeling probe bursting brain activity at different timescales
Source: Neuroimage. 2022 Feb 15;247:118850. doi: 10.1016/j.neuroimage.2021.118850 (PMC8803543; doi:10.1016/j.neuroimage.2021.118850)
Supplement: Supplementary file 1 [file mmc1.pdf]

Microstates and power envelope hidden Markov modeling probe bursting brain activity at different timescales

Supplementary material

Coquelet N[1], De Tiège X[1,2], Roshchupkina L[1,3], Peigneux P[3], Goldman S[1,2], Woolrich M[4] & Wens V[1,2]

[1] Laboratoire de Cartographie fonctionnelle du Cerveau (LCFC), UNI – ULB Neuroscience Institute, Université libre de Bruxelles, Brussels, Belgium.

[2] Magnetoencephalography Unit, Clinics of Functional Neuroimaging, Service of Nuclear Medicine, CUB – Hôpital Erasme, Brussels, Belgium.

[3] Neuropsychology and Functional Neuroimaging Research Unit (UR2NF), Centre for Research in Cognition and Neurosciences (CRCN), UNI – ULB Neuroscience Institute, Université libre de Bruxelles (ULB), Brussels, Belgium.

[4] Oxford Centre for Human Brain Activity, Wellcome Centre for Integrative Neuroimaging, Department of Psychiatry, University of Oxford, Oxford, United Kingdom.

S1. Effect of increased wideband filter on microstates

The EEG microstate literature commonly relies on a frequency range that includes delta rhythms. We repeat our main microstate analysis (K = 4 AAHC at GFP peak time points, frequency sampling at 40 Hz) but from EEG/MEG signals filtered between 1 Hz and 40 Hz.

Figure S1 shows the spatial topographies of EEG and MEG microstates derived both between 4–30 Hz and 1–40 Hz. We observe a high spatial correspondence among pairs of microstates, and in fact the lowest spatial correlation among these pairs was. This indicates that widening the frequency range has no effect on the spatial signature of microstates.

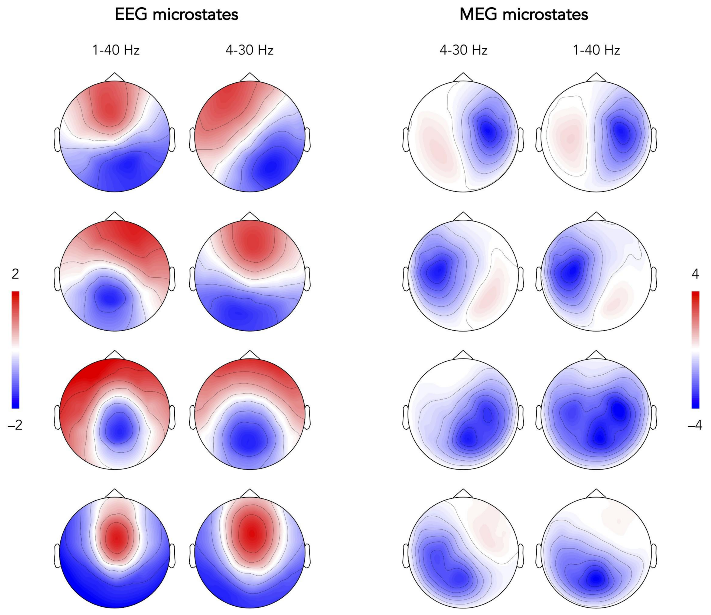

Figure S1: Effect of the filter settings (1–40 Hz vs. 4–30 Hz) on the spatial signature of EEG (**left**) and MEG (**right**) microstates (four-cluster AAHC at GFP peaks). Microstates were paired based on their unambiguous spatial correspondence.

On the other hand, widering the frequency range from 4–30 Hz to 1–40 Hz significantly increased the mean lifetime of microstates estimated from their (non-smoothed) activation time series ($t_{41} > 34.8$, $p = 0$; compare Table S1 to Table 1 in the main text).

EEG microstates			MEG microstates		
	Mean lifetimes (ms)	Fractional occupancies (%)		Mean lifetimes (ms)	Fractional occupancies (%)
Microstate A	55 ± 7	23.3 ± 3.9	Microstate α	37 ± 2	11 ± 1.6
Microstate B	57 ± 6	24.9 ± 4.3	Microstate β	42 ± 2	16.2 ± 2.2
Microstate C	58 ± 6	26.4 ± 3.2	Microstate γ	102 ± 9	62.3 ± 2.3
Microstate D	55 ± 4	25.3 ± 3.9	Microstate δ	37 ± 2	10.3 ± 1.9

Table S1: Mean lifetimes and fractional occupancies (mean ± SD) associated with each microstate inferred from EEG or MEG topographies filtered between 1–40 Hz (without temporal smoothing on microstate activation time series).

S2. Comparison of "two-level" and "group-level" microstate clustering

We repeat here the main microstate analysis ($K = 4$ AAHC of sensor maps downsampled at 40 Hz and restricted to time points of locally maximal GFP), with the only difference that the clustering is directly performed at the group level (i.e., after concatenation of EEG/MEG signals across all subjects), which is more comparable to the group HMM analysis than the two-step microstate clustering presented in the main text.

Figure S2 displays pairs of microstates obtained in both cases and shows that this methodological detail does not impact microstate topographies (spatial correlation among pairs: $R > 0.71$). Mean lifetimes and fractional occupancies were not significantly affected either ($t_{41} < 1.8$, $p > 0.08$ for mean lifetime; $t_{41} < 1.14$, $p > 0.26$ for mean fractional occupancy).

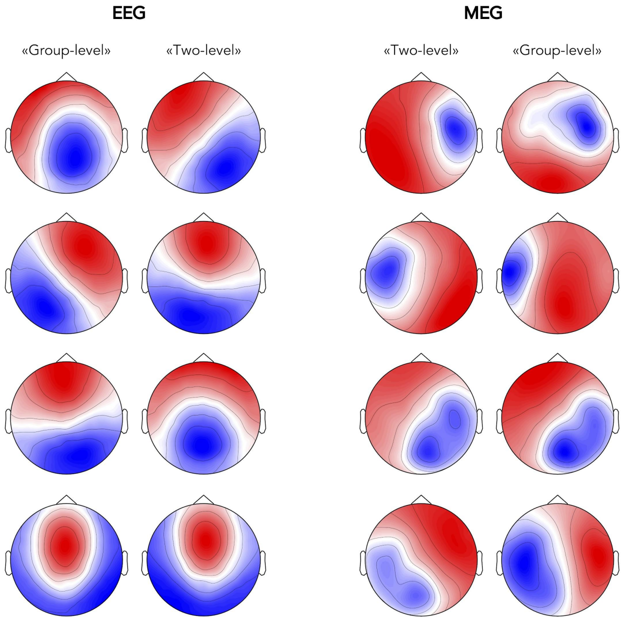

Figure S2: Effect of clustering type (“group-level” vs. “two-level”) on the spatial signature of EEG (**left**) and MEG (**right**) microstates (four-cluster AAHC at GFP peaks, 40 Hz sampling rate). Microstates were paired based on their unambiguous spatial correspondence.

S3. Microstate clustering without temporal restriction

In the main text, microstate topographies were inferred from local GFP maxima, whereas the HMM ran over the continuous signals. We repeat here the main microstate analysis ($K = 4$ AAHC of sensor maps at 40 Hz sampling rate) but without restriction to isolated time points.

Figure S3 shows that microstate topography is qualitatively unaffected by this methodological difference. As discussed in the main text, this is because the AAHC algorithm explicitly biases microstates towards maximal GFP, suggesting that microstates are mostly sensitive to neural activity around GFP peak times. In line with this suggestion, we observed an excellent one-to-one temporal correspondence between each pair of microstates (Fig. S3).

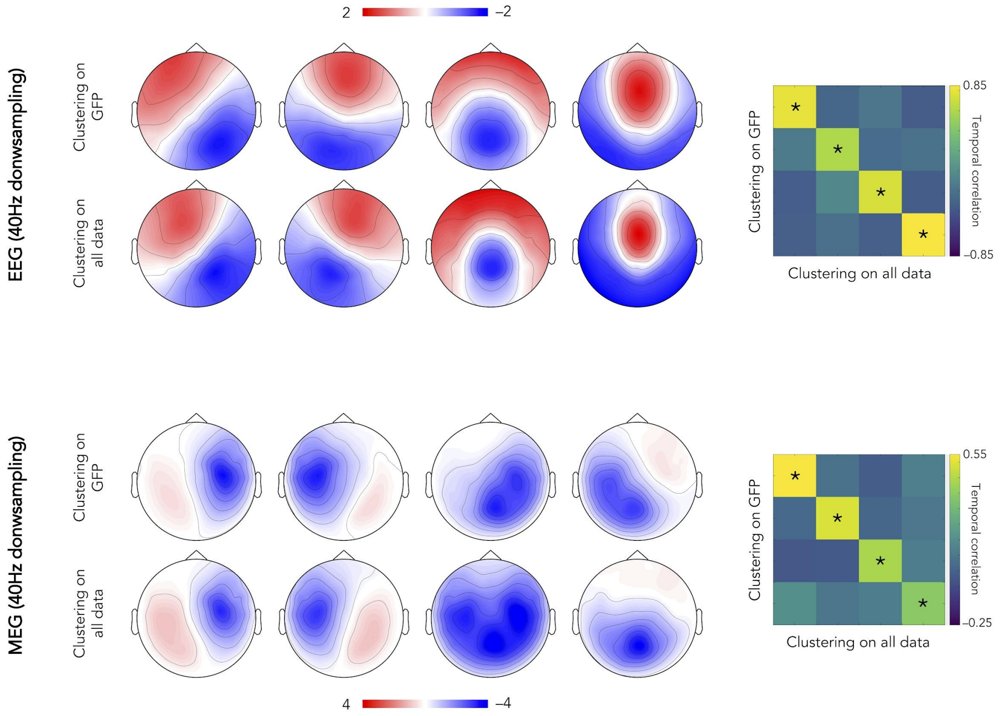

Figure S3: Effect of the temporal restriction to GFP local maxima on EEG (**top**) and MEG (**bottom**) microstates (four-cluster AAHC, 40 Hz sampling rate). Microstates were paired

modality based on their unambiguous spatial correspondence. Temporal correlations of microstate activation time series (without temporal smoothing) are shown on the far right.

S4. Effect of temporal smoothing on microstate activation time series

The EEG microstate literature commonly employs an ad-hoc temporal smoothing on the microstate activation time series. We repeated our microstate analysis (i.e., $K = 4$ AAHC at GFP peak time points, sampling frequency at 40 Hz) including a temporal smoothing of microstate activation time series. Microstate topographies were by construction identical to those shown in Fig. 3, but temporal smoothing significantly increased microstate mean lifetimes ($t_{41} > 49.8$, $p = 0$; compare Table S2 to Table 1 in the main text).

EEG microstates			MEG microstates		
	Mean lifetimes (ms)	Fractional occupancies (%)		Mean lifetimes (ms)	Fractional occupancies (%)
Microstate A	138 ± 19	28 ± 6.5	Microstate α	102 ± 7	18.6 ± 3.1
Microstate B	122 ± 24	24.8 ± 6.8	Microstate β	117 ± 12	26.7 ± 5.4
Microstate C	124 ± 26	24.1 ± 7	Microstate γ	141 ± 12	37.5 ± 4.3
Microstate D	122 ± 23	23 ± 6.3	Microstate δ	94 ± 9	17.2 ± 2.9

Table S2: Mean lifetimes and fractional occupancies (mean ± SD) associated with each microstate inferred from EEG or MEG topographies with temporal smoothing on microstate activation time series.

Figure S4 shows the spatio-temporal correlation analysis between temporally-smoothed microstates and HMM states. Results were virtually identical to those obtained with raw

(non-smoothed) microstate activation time series (compare with Fig. 5). This shows that temporal smoothing of microstate activations does not impact our main results.

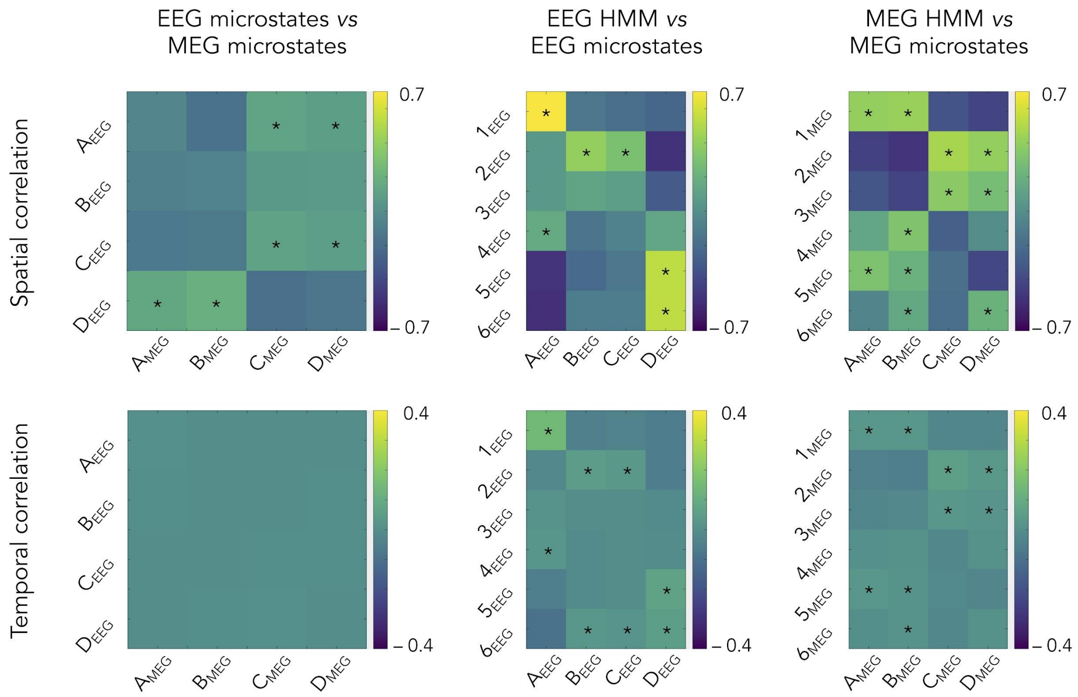

Figure S4: Spatial and temporal correlations in the case of smoothed microstate activation time series. Compare with Fig. 5 in the main text.

S5. Hidden Markov modeling of EEG power envelopes at higher dimensionality

In our main HMM analyses, we fixed the data dimensionality (i.e., the number N of principal components fed to the HMM classification algorithm) so the fraction of explained variance is identical for MEG and EEG power envelope signals. This approach naturally takes into account the inherently distinct spatial smoothness of the two recording modalities. However, this difference might impact HMM state classification in EEG and underlie some discrepancies with MEG HMM states. Here, we consider the HMM analysis of EEG power envelopes with $N = 41$, as for MEG.

Figure S5 shows side-by-side the spatial signature of EEG power envelope HMM states at low ($N = 10$; see Fig. 4, left) and higher ($N = 41$) dimensions. Sensor-level maps show that HMM states are qualitatively similar, although some brain power maps exhibited a higher degree of bilaterality (see, e.g., state 1) and more dynamical competition (state 2) when $N = 41$. State 5 was the only state qualitatively different as it involved a frontal activation at when $N = 41$ instead of a left sensorimotor activation at when $N = 10$.

Figure S6 shows the spatial (Fig. S6, top) and temporal (Fig. S6, bottom) correlation analyses between EEG HMM states inferred at $N = 41$ and MEG HMM states at the same dimensionality (Fig. S6, left) or EEG microstates (Fig. S6, right). Despite some qualitative topographical differences, increasing the dimensionality of EEG power envelope data did not affect substantially the spatial or temporal correspondence across state clustering methods or recording modality (compare to Fig. 5). The observations and conclusions discussed in the main text thus stand.

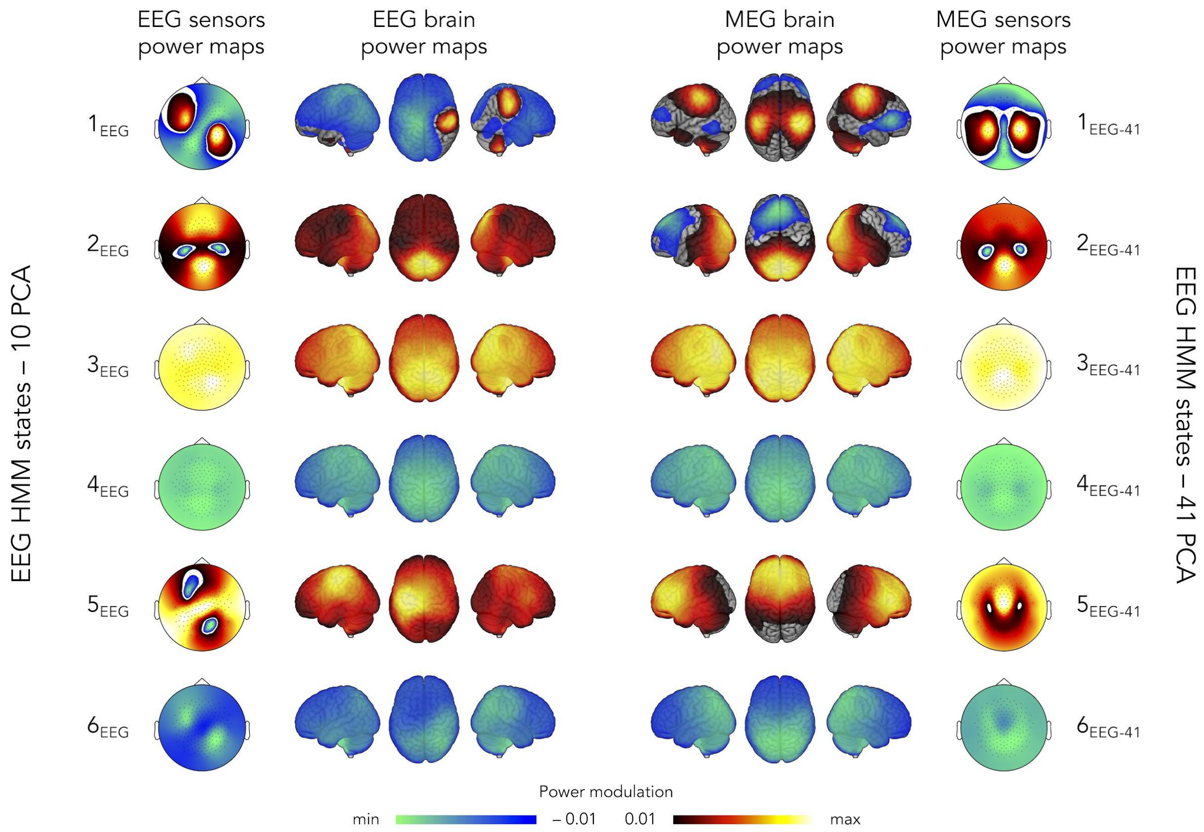

Figure S5: Spatial signature of EEG sensor-level power envelope HMM states obtained with $N =$ 10 (**left;** see Fig. 4, left in the main text) and $N = 41$ (**right**) components retained prior to HMM inference.

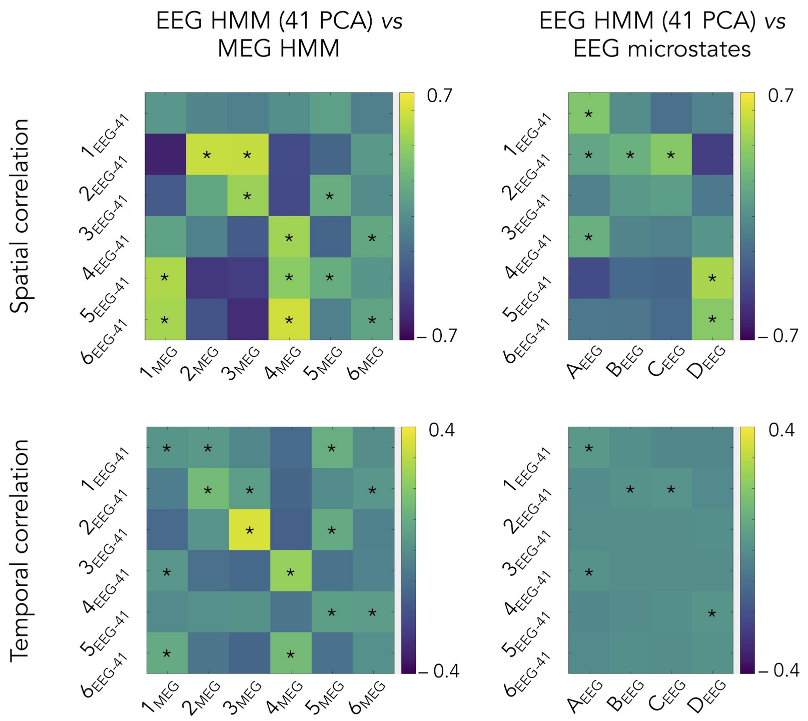

Figure S6: Spatial (**top**) and temporal (**bottom**) correlations when the HMM of EEG power envelopes is inferred from $N = 41$ components. Compare with Fig. 5 in the main text.

S6. Power envelope hidden Markov model with four states

We repeated the sensor-level power envelope HMM analysis by lowering the number of states to classify from $K = 6$ (used in the main text) to $K = 4$, for better comparability with the four-microstate clustering.

Figures S7 and S8 present the main results of this analysis. The four-state HMM merely disclosed a subset of the six-state HMM, specifically states 2_{MEG}–5_{MEG} for MEG and states 1_{EEG}, 3_{EEG}–5_{EEG} for EEG (compare Fig. S7 to Fig. 4 in the main text). The spatio-temporal correlation analysis comparing microstates vs. HMM states and EEG vs. MEG (Fig. S8) then naturally led to similar observations than in the case of six-state HMM (compare to Fig. 5 in the main text). Therefore, the observations and conclusions discussed in the main text stand.

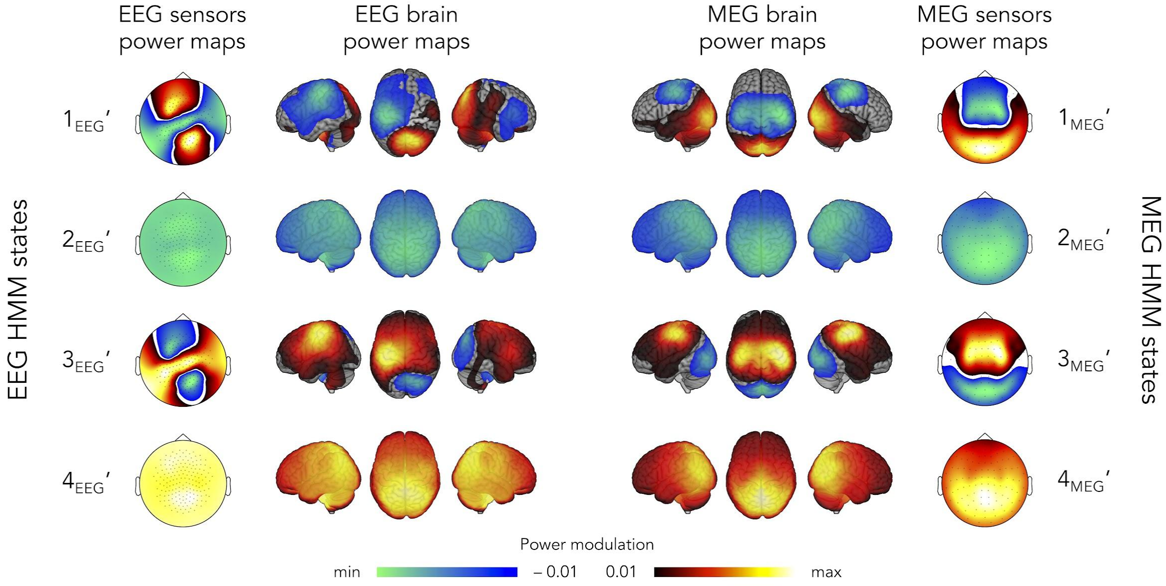

Figure S7: Spatial signature of EEG (**left**) and MEG (**right**) sensor-level power envelope HMM states obtained with $K = 4$. Compare with Fig. 4 in the main text.

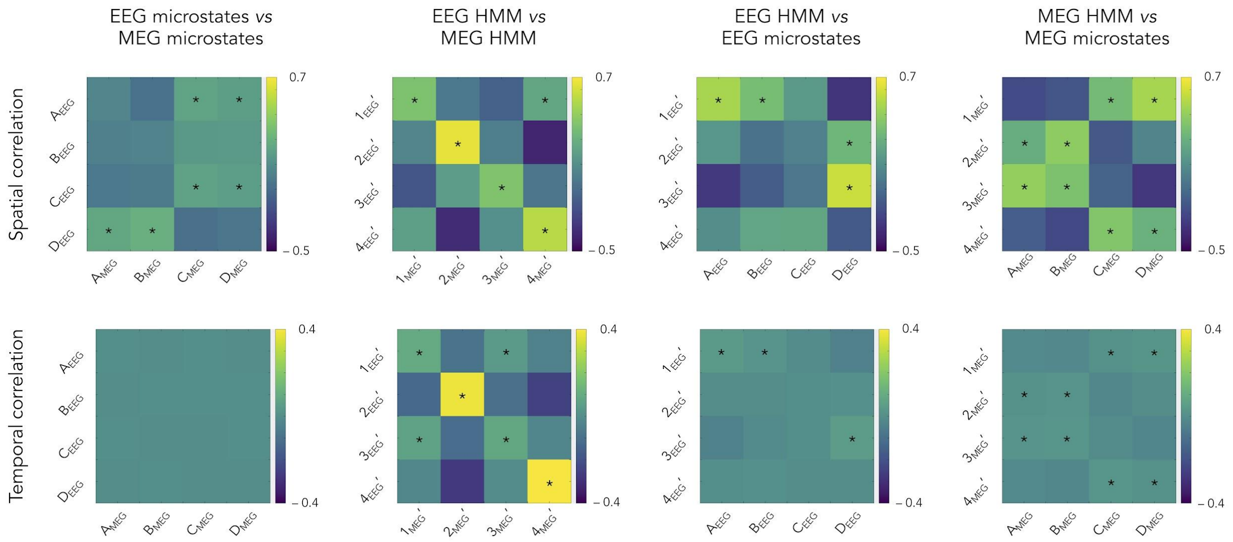

Figure S8: Spatial (**top**) and temporal (**bottom**) correlations in the case of a four-state power envelope HMM. Compare with Fig. 5 in the main text.

S7. Microstate clustering with six states

We repeated the microstate analysis with $K = 6$ clusters, and examined to what extent this affects our results and comparison to the six-state power envelope HMM.

Figure S9 shows that the four canonical microstates found in Fig. 3 (see main text) are recovered among the six resulting microstates denoted A_{EEG}'–F_{EEG}' and A_{MEG}'–F_{MEG}'. For EEG, microstate E_{EEG}' was spatially similar to microstate D_{EEG}' (Fig. S9), suggesting that the increase in number of clusters led to a splitting of microstate D_{EEG} (Fig. 3). On the other hand, microstate F_{EEG}' appeared new. Interestingly, for MEG, the two extra microstates E_{MEG}' and F_{MEG}' did not bring new significant information at the level of power modulations, since their brain power maps did not reach the statistical threshold .

Additionally, microstates were slightly shorter lived when increasing the number of microstates (EEG: mean ± SD: 33 ± 1 ms; range : 31 – 34 ms; MEG: mean ± SD: 33 ± 5 ms; range : 27 – 40 ms), and displayed a higher percentage of global variance explained (EEG: 61%; MEG: 37%) compared to the four-microstate analysis.

Figure S10 summarizes the spatio-temporal comparison of the six-microstate clustering vs. the six-HMM states (described in the main text, see also Fig. 5). The observations and conclusions discussed in the main text appeared to remain valid.

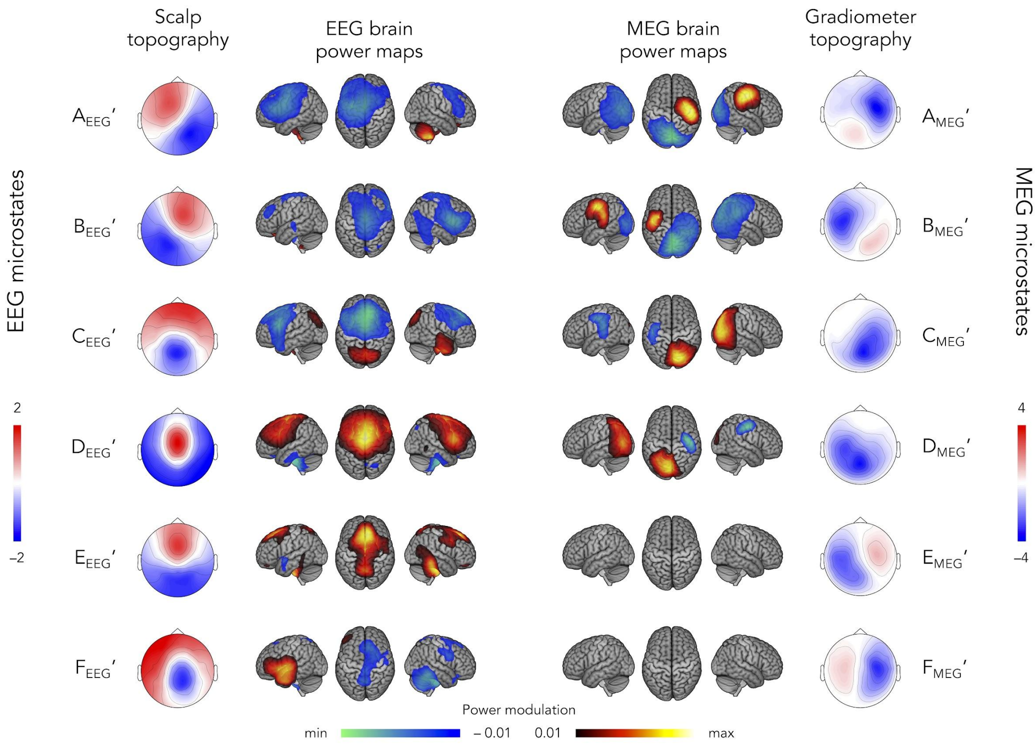

Figure S9: Spatial signature of EEG (**left**) and MEG (**right**) microstates obtained with $K = 6$. Compare with Fig. 3 in the main text.

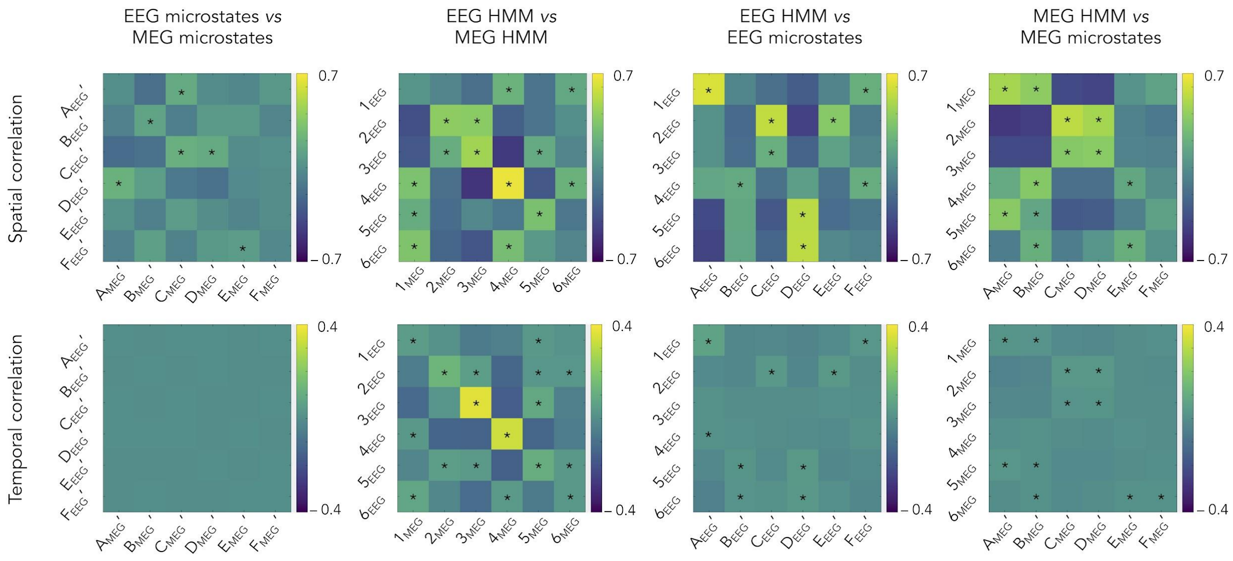

Figure S10: Spatial (**top**) and temporal (**bottom**) correlations in the case of a six-microstate clustering. Compare with Fig. 5 in the main text.
